# Cell wall modulation by drought and elevated CO_2_ in sugarcane leaves

**DOI:** 10.3389/fpls.2025.1567201

**Published:** 2025-04-30

**Authors:** Alexandre Junio Borges Araujo, Amanda Pereira de Souza, Débora Pagliuso, Mauro de Medeiros Oliveira, Bruno Viana Navarro, Adriana Grandis, Marcos Silveira Buckeridge

**Affiliations:** Laboratório de Fisiologia Ecológica de Plantas, Departamento de Botânica, Instituto de Biociências, Universidade de São Paulo, São Paulo, SP, Brazil

**Keywords:** grasses, climate change, abiotic stress, transcriptome, glycosyltransferases, NDP-sugar

## Abstract

Climate change poses significant challenges to global agriculture, with elevated atmospheric CO2 (eCO_2_) concentrations and increased frequency of droughts affecting crop productivity. Understanding how economically important crops like sugarcane respond to these combined stresses is essential for developing resilient cultivars. This study explores the effects of eCO_2_ and drought stress on sugarcane growth and cell wall composition. Sugarcane plants were cultivated under CO_2_ treatments (390 ppm and 780 ppm) and subjected to drought stress. Leaf biomass, cell wall composition, and global transcriptome sequencing were analyzed. eCO_2_ (780 ppm) significantly increased leaf biomass by 64%, attributed to enhanced photosynthesis and water-use efficiency. Conversely, drought reduced leaf biomass by 45%, highlighting sugarcane’s sensitivity to water scarcity. When both conditions were combined, eCO_2_ mitigated drought’s negative impact, maintaining biomass at levels comparable to ambient conditions. Despite notable changes in biomass, cell wall biomass was only slightly affected. Under drought, a 14% reduction in cell wall biomass was observed alongside compositional changes, including reduced arabinosylation in glucuronoarabinoxylan (GAX). This alteration, supported by decreased xylan arabinosyl transferase (XAT) expression and reduced arabinose content, suggests stronger associations between GAX and cellulose, potentially enhancing drought tolerance by modifying cell wall rigidity and flexibility. Under eCO_2_, cell wall composition was altered, with reductions in glucose and uronic acid in specific fractions, indicating decreased mixed-linkage glucan (MLG) and pectin. These changes likely increased cell wall flexibility, supporting rapid growth. Combined eCO_2_ and drought treatments amplified specific modifications, such as enhanced fucosylation of xyloglucan (XG) and potential MLG expansion, both linked to stress adaptation. Overall, the findings underscore the critical role of cell wall plasticity in sugarcane’s response to abiotic stress. While eCO_2_ boosted growth and partially alleviated drought effects, structural changes in cell wall composition under these conditions further contribute to stress resilience, emphasizing the adaptive mechanisms of sugarcane to environmental challenges. This is the first report in which eCO_2_, and drought are combined to evaluate the response of sugarcane to the impact of climate changes.

## 1 Introduction

Sugarcane (*Saccharum* spp.) is one of the most economically significant crops due to its versatile raw material applications (Leão, 2002). Brazil is the largest producer, accounts for 46% of global production and primarily cultivates sugarcane for sugar and bioethanol, a key alternative to fossil fuels (Bordonal et al., 2018; Grandis et al., 2024). This makes sugarcane one of Brazil’s most important crops. However, its production is increasingly threatened by climate change, particularly rising CO_2_ levels and more frequent droughts, which impact yield and sustainability (Da Cruz and MaChado, 2023). The interaction between elevated CO2 (eCO_2_) and drought stress remains complex and not fully understood. Studying these factors together is crucial, as their combined effects may differ from their individual impacts. Developing mitigation strategies through advanced crop technologies requires a comprehensive approach that considers both stressors to ensure long-term productivity and resilience.

The interaction between plants and eCO_2_ has been widely studied across various crops, with responses varying depending on species and environmental conditions (Arenque et al., 2014; Liu et al., 2024). Generally, eCO_2_ benefits most plants by improving water use efficiency and photosynthesis rates, leading to enhanced biomass production (Fortirer et al., 2023). In C4 plants, maize and sorghum, typically show a limited response to eCO_2_ (Leakey et al., 2006; Ottman et al., 2001), while sugarcane has exhibited similar benefits, resembling the response observed in C3 plants productivity improvement biomass and sugar accumulation (De Souza et al., 2008).

Including increase in atmospheric CO_2_, climate change is driving a range of extreme effects, including altered precipitation patterns that increase the frequency of droughts events in Central and South America (Intergovernmental Panel on Climate Change (IPCC), 2022). Drought stress is well-documented for its negative impact on plant growth and productivity across many crops (Yang et al., 2021; Kapoor et al., 2020). In sugarcane, while moderate drought stress during maturation can enhance sucrose accumulation, severe drought triggers anatomical, physiological, and cellular changes that hinder plant development (Dlamini, 2021), leading to a complex regulation mechanism in these plants. Several changes in metabolic mechanisms occur in both intensities, including changes in photosynthesis rate, prevention of cell dehydration, mitigation of protein damage, and structural modifications in the cell wall (Waraich et al., 2011; Delmer et al., 2024).

The plant cell wall is a complex network of glycoproteins, lignin, and polysaccharides, including cellulose, hemicelluloses, and pectins (Delmer et al., 2024). Cellulose consists of large β-1,4-glucan chains organized into microfibrils connected by hemicelluloses and embedded within a pectic matrix. Hemicelluloses comprise branched and unbranched chains of glucans, mannans, and xylans (Cosgrove and Jarvis, 2012; Delmer et al., 2024). Pectin includes a backbone of galacturonic acids that form homogalacturonan (HG) and rhamnogalacturonan II (RG-II), and a backbone of galacturonic acids and rhamnose which form rhamnogalacturonan I (RG-I) (Mohnen et al., 2008).

The structure of cell walls is a result of a Glycomic Code, which represents the organization of cell wall polysaccharides within the walls, giving rise to different combinations of cell wall domains (cellulose-hemicellulose-lignin, the pectin, and the structural protein domains) (Buckeridge, 2023). These domains characterize three cell wall types across higher plant clades according to the primary cell wall (McCann, 1992; Carpita and Gibeaut, 1993; Silva et al., 2011). In sugarcane, the cell wall is classified as Type II, which displays walls containing a higher proportion of glucuronoarabinoxylan (GAX) and β-1,3-1,4-mixed-linked glucan (MLG). As a result, another feature distinguishing Type II from Types I and III walls is the relatively lesser amounts of xyloglucan (XG), pectins, and lignin (De Souza et al., 2013). A common feature of the three wall types, as viewed by the biosemiotics approach of the Glycomic Code (Buckeridge, 2023), is that the three wall types provide the biomechanical properties necessary for plant development and adaptation to both biotic and abiotic stresses (Le Gall et al., 2015; Delmer et al., 2024).

**Figure 1:**
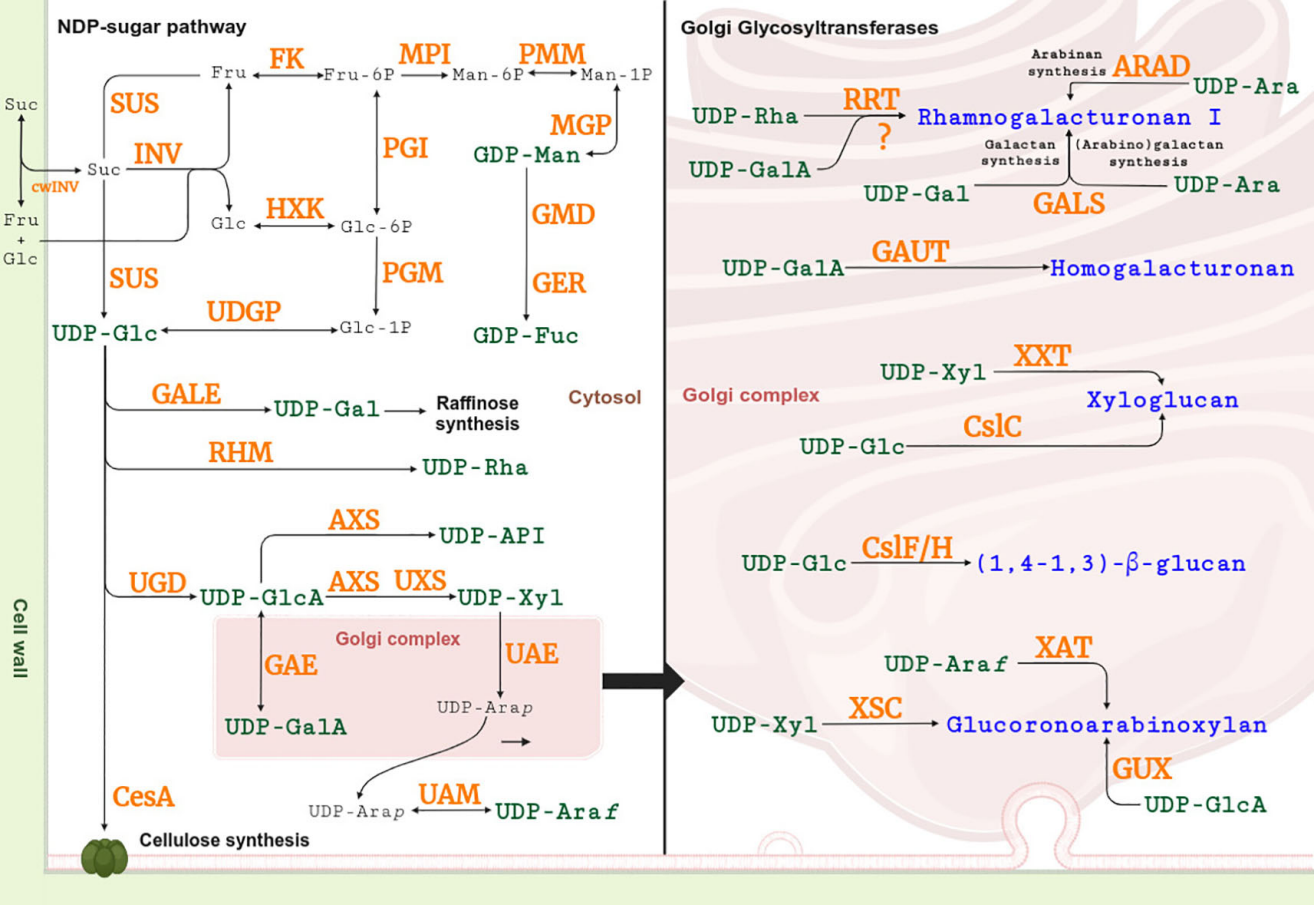
NDP-sugar synthesis and cell wall polysaccharide assembly pathway. Enzymes are indicated in orange. The NDP-sugar (green) pathway centers on UDP-glucose (UDP-Glc). Excluding GDP-fucose (GDP-Fuc) and GDP-mannose (GDP-Man), UDP-Glc is used to generate the other seven NDP-sugars: UDP-galactose (UDP-Gal), UDP-rhamnose (UDP-Rha), UDP-glucuronic acid (UDP-GlcA), UDP-galacturonic acid (UDP-GalA), UDP-apiose (UDP-Api), UDP-xylose (UDP-Xyl), UDP-arabinopyranose (UDP-Ara*p*), and UDP-arabinofuranose (UDP-Ara*f*). UDP-Glc can synthesize cellulose in the cell membrane or be sent to the Golgi apparatus along with the other NDP-sugars to synthesize pectins and hemicellulose (blue). For detail of the abbreviations and references, see **Supplementary Data Sheet 1** and **Supplementary Data 1**. Adapted from Pagliuso et al. (2022) and Verbančič et al. (2018).

The polysaccharides synthesis involves numerous gene families and occurs in two distinct stages. Initially, the production of most monosaccharides (nucleotide diphosphate sugars, NDP-sugars) takes place in the cytosol and Golgi complex, relying on UDP-glucose derived from sucrose and glucose metabolism, while GDP-mannose and GDP-fucose are synthesized from fructose-6-phosphate (**Figure 1**) (Verbančič et al., 2018). Subsequently, polysaccharides are assembled by glycosyltransferases (GTs), with cellulose synthesis occurring at the plasma membrane and the assembly of pectins and hemicelluloses occurring in the Golgi complex (**Figure 1**) (Mohnen et al., 2008; Hansen et al., 2012). In contrast, polysaccharide hydrolysis is mediated by glycosyl hydrolases in the apoplastic matrix (Gilbert, 2010).

The cell wall structure is influenced by modifications in response to eCO_2_ and drought independently depending on plant species and duration of exposure. The high CO_2_ concentration generally reduces cell wall thickness, enhancing cell wall loosening and elasticity, which facilitates cell expansion (Taylor et al., 1994; Pérez-López et al., 2010; Kim et al., 2015). In contrast, under low water availability, cell expansion is inhibited, leading to smaller plants with thicker cell walls, promoting modifications to prevent water loss (Leucci et al., 2008; Tardieu et al., 2014; Balducci et al., 2015). Molecular analyses support these findings, showing a regulation in the gene expression related to the synthesis and hydrolysis of cell wall polysaccharides (May et al., 2013; Le Gall et al., 2015). Despite these observations, the effect of eCO_2_ and drought in cell walls remains scarce, even though this combination is likely representative of future climate change scenarios.

It is well known that the cell wall undergoes modifications in response to stress. Due to the intensification of climate change and the combined effects of drought, temperature, and CO_2_ concentrations, it is crucial to evaluate the sugarcane’s responses to guarantee more knowledge and mitigate the future effects. Therefore, this study aimed to investigate how sugarcane cell wall synthesis is affected by drought, eCO_2_, and combined eCO_2_+drought treatment by molecular, biochemical, and physiological approaches.

## 2 Materials and methods

### 2.1 Plant material and experimental design

Sugarcane (*Saccharum* spp.) from the Brazilian commercial variety SP80-3280 was used in this study. The experiment was conducted in the summer of 2011 at the University of São Paulo, Brazil (23°33'55"S, 46°43'51" W). Culms were sectioned into single-node segments containing vegetative buds and planted in Plantmax® substrate, which consists of pine chips, vermiculite, and peat. Plants were established for 10 days and then transplanted into a 15L pot with the same substrate.

**Figure 2:**
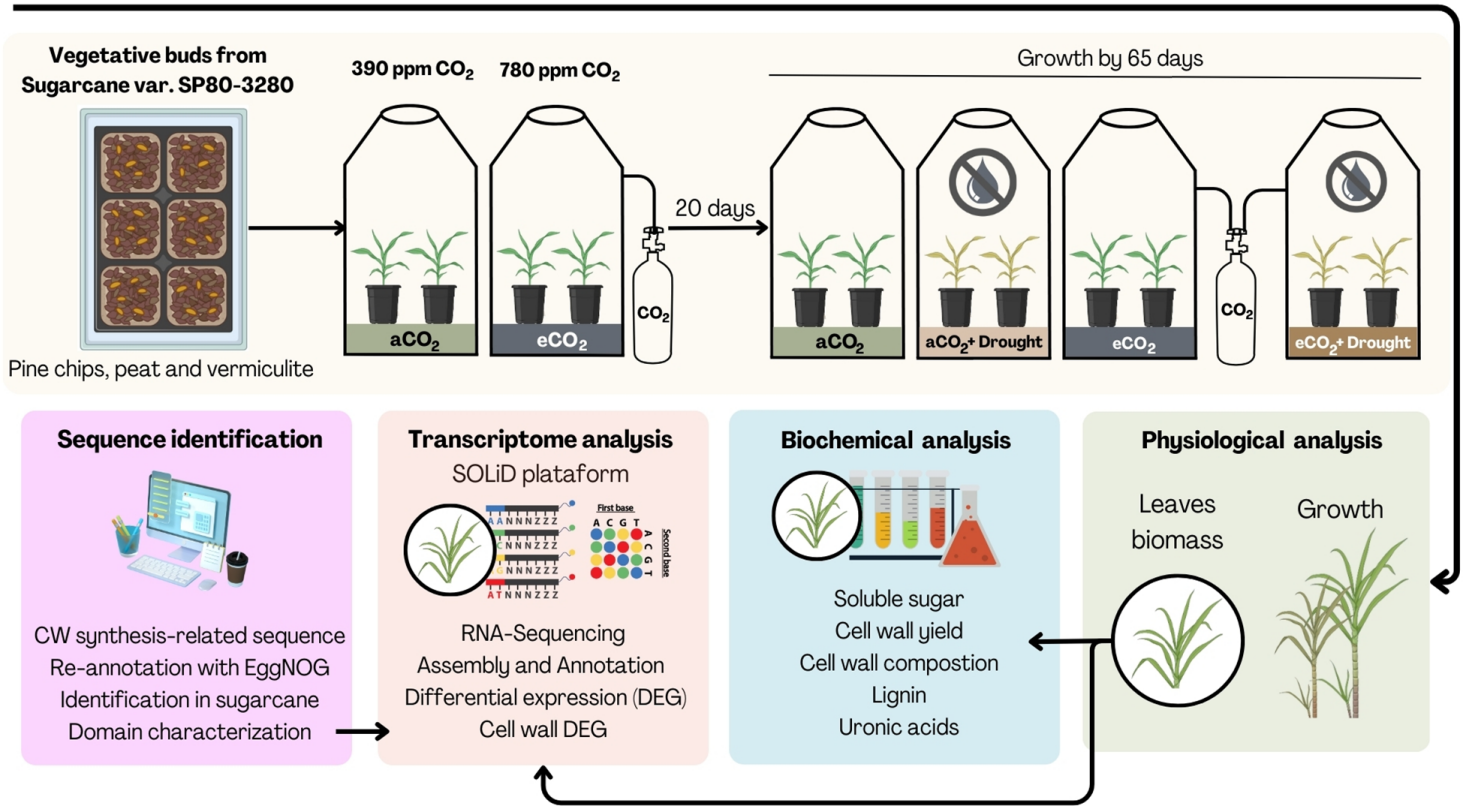
Experimental design used to evaluate the response of sugarcane cell walls under different CO_2_ concentrations and watering conditions. Sugarcane plants (var. SP80-3280) were grown in Open-Top Chambers (OTCs) under four treatment conditions: ambient CO_2_ ([aCO_2_], 390 ppm) with adequate watering, elevated CO_2_ ([eCO_2_], 780 ppm) with adequate watering, ambient CO_2_ with drought stress [Dro], and elevated CO_2_ with drought stress [eCO_2_+Dro]. Growth parameters, cell wall fractionation, and transcriptome analysis were performed to assess the impact of these conditions on the sugarcane cell wall.

The pots with plants were randomly distributed into four open-top chambers (OTCs) 130 cm in diameter and 300 height. Two OTCs were maintained at 390 ppm CO_2_ ([aCO_2_]), and two at 780 ppm CO_2_ ([eCO_2_]). After 20 days under these conditions, one OTC in [aCO_2_] and one in [eCO_2_] were subjected to drought stress by suspending watering: [aCO_2_+Dro] and [eCO_2_+Dro]. Plants were rotated weekly within the OTCs, and every 15 days, they were switched between OTCs to avoid acclimation effects. Fertilization was performed using an N:P:K (18:00:27). After 65 days of cultivation, leaves were harvested for biomass, cell wall composition, and gene expression analysis. The experimental design is summarized in **Figure 2**.

### 2.2 Global transcriptome sequencing

The leaf+1 was frozen in liquid nitrogen, ground, and extracted in 1.5 mL TRIzol (Invitrogen). The acquired RNA was purified using the PureLink RNA Mini Kit (Ambion, Life Technologies) with the manufacturer's instructions. RNA integrity was confirmed via electrophoresis on a 0.8% (w/v) agarose gel, and the quantity and purity were assessed using a NanoDrop NDC1000 UV–VIS Spectrophotometer (Thermo Scientific), with samples considered as purity showing 260/280 nm and 260/230 nm ratios between 1.8–2.2 and 1.6–2.2. Ribosomal RNA (rRNA) was removed using the RiboMinus Plant Kit and concentrated with the RiboMinus Concentration Module (Invitrogen), according to the manufacturer's instructions. rRNA contamination and RNA quantification were verified using a Bioanalyzer 2100 (Agilent Technologies).

cDNA libraries and sequencing following the manufacturer's instructions (Life Technologies). The theoretical coverage was 80X, using a sequencing Flowchip for each set of 12 samples. Sequencing was performed on the SOLiD platform, with quality monitored using the Applied Biosystems SOLiD 4 System SETS software. For expression analysis, only lanes with a percentage of good/best beads greater than 50% in all run cycles were considered.

### 2.3 Transcriptome construction, assembly, and annotation

All RNA-Seq samples were obtained with > 120 million raw reads. Subsequently, the trimming was performed using Trimmomatic v0.38 (Bolger et al., 2014) with a sliding window quality cut-off of Q>20 and a minimum read size of 60 base pairs. Adapter sequences and ribosomal subunits were removed using Trimmomatic and SortMeRNA v4.3.6 (Kopylova et al., 2012). Read quality before and after treatment was assessed with FastQC v0.11.8 (de Sena Brandine and Smith, 2021). The transcriptome was assembled by mapping reads to the sugarcane SP80-3280 reference genome (Souza et al., 2019) using BWA v0.7.17 (Li and Durbin, 2009). Alignment outputs were converted to BAM format with Samtools v1.7 (Li et al., 2009) and used as input for StringTie v1.3.0 (Pertea et al., 2016) to generate transcript sequences. Functional annotation was performed using eggNOG-mapper v2.1.6 (Cantalapiedra et al., 2021), which provides essential information such as orthologs, domain identification, and associations with Gene Ontology terms and metabolic pathways. Input files were translated into protein sequences (CDS) using CodAn (Nachtigall et al., 2021). EggNOG v5.0.2, UniProt (The UniProt Consortium, 2021), Pfam (Mistry et al., 2021), and Kyoto Encyclopedia of Genes and Genomes (KEGG) (Kanehisa and Goto, 2000) were used to ensure annotation quality.

After sequencing, strict quality control was applied to ensure accuracy, with only 50% of the sequences reaching the required standards. The reads were then mapped against the genome of the same sugarcane variety used in this study (*Saccharum* spp. var SP80-3280 - Souza et al., 2019), resulting in an identification of a total of 391,000 transcripts, of which 36,608 transcripts were expressed among the replicates.

### 2.4 Identification of sugarcane cell wall polysaccharide synthesis-related transcripts

De Oliveira et al. (2022), and Partida et al. (2025) adapted the sequence identification methodology to identify sequences in polyploid sugarcane. NDP-sugar glycosyltransferase (GT) sequences were retrieved from the references in **Supplementary Table 1**. KEGG IDs (https://www.genome.jp/kegg/) and protein names were used as queries to extract sequences from *Oryza sativa and S. bicolor* in Phytozome v13 (Goodstein et al., 2012), leveraging their phylogenetic proximity to sugarcane. These proteins were subsequently used to obtain gene ontology (GO) annotations through EggNOG v4.5.1 (Huerta-Cepas et al., 2016). Using the associated GO terms, sequences in sugarcane were identified from the annotated genome of SP80-3280 variety (Souza et al., 2019).

The SP80-3280 protein sequences were analyzed for domain architecture using HMMER version 2.41.2 (Potter et al., 2018) to ensure functional relevance and exclude non-functional sequences. Sequences containing canonical domains with at least 70% domain coverage were selected for subsequent expression analysis.

### 2.5 Expression analysis

Previously identified sequences were used as queries to assess transcript expressions, with only those expressed in at least two replicates included in the analysis. Expression patterns were measured as counts per million (CPM) and visualized using heatmaps generated in MetaboAnalyst software (Pang et al., 2024), applying default parameters and treatment-level normalization.

The differential expression among treatments was analyzed using a general linear model (GLM) due to the data’s non-normality (Liu et al., 2019). A gamma distribution was applied to the GLM to account for the positively skewed CPM data. Tukey's test was followed with adjusted p-values to control for multiple testing and determine group separations. The GLM and statistical tests were implemented in R software version 4.2.2 (R Core Team, 2021). For data visualization, a log10 transformation in CPM was used to generate heatmaps of differentially expressed (DE) transcripts, using MetaboAnalyst 6.0 (Pang et al., 2024).

### 2.6 Cell wall preparation and fractionation

Total leaves were dried at 60 °C and ground in a ball mill until a fine and homogeneous powder was obtained. A 500 mg sample of the ground leaves was extracted five times with 80% (v/v) ethanol at 80 °C for 20 minutes to remove soluble sugars and other soluble compounds following De Souza et al. (2013). Subsequently, alcohol insoluble residues (AIR) were extracted twice in 90% (v/v) dimethyl sulfoxide (DMSO) for 1 hour each to remove starch content, followed by an extraction for 12 hours to ensure complete removal (Carpita, 1983). The de-starched AIR, or cell wall, was recovered after centrifugation, washed with water, and freeze-dried.

Cell wall fractionation was performed as described by De Souza et al. (2013) with sequential extractions to solubilize each class of polysaccharides. The cell wall was extracted three times 0.5% (w/v) ammonium oxalate at 80 °C with continuous mixing for 1 hour to remove pectins. The supernatants were recovered by centrifugation, and the oxalate-extracted residues were further extracted with 3% sodium chlorite in 0.3% (v/v) acetic acid at 70 °C for 3 hours to remove lignin. The supernatants were recovered, and the hemicelluloses from the sodium chlorite-treated cell wall residues were extracted three times with 4 M sodium hydroxide (NaOH) and 3 mg/mL of sodium borohydride at room temperature for 1 hour each. All the supernatants were recovered, neutralized, dialyzed, and freeze-dried. The residues were washed and freeze-dried to calculate the mass balance yield.

### 2.7 Neutral and acid monosaccharide analysis

For each cell wall fraction, 2 mg were hydrolyzed with 2N trifluoroacetic acid (TFA) at 100 °C for 2 hours, vacuum-concentrated, and resuspended in 1 mL of Milli-Q pure water. Additionally, the residue fraction was hydrolyzed with 72% (v/v) sulfuric acid (H_2_SO_4_) at 30 °C for 45 minutes, followed by diluting the acid to 4% and incubating at 100 °C for 1 hour. Hydrolyzed material was analyzed using High-Performance Anion-Exchange Chromatography with Pulsed Amperometric Detection (HPAEC-PAD) on a CarboPac SA10 column, integrated into the ICS 5000 system from Dionex® for the identification and quantification of the cell wall monosaccharides. The column was eluted isocratilly mixture of 99.2% water and 0.8% (v/v), flowing at 1 mL.min^-1^, and were detected using a post-column base containing 500 mM NaOH (0.5 mL/min). The cell wall monosaccharides were determined using a standard curve.

For uronic acid content analysis, 5 mg from AIR and cell wall fractions were hydrolyzed with H_2_SO_4_ 99.8% in an ice bath for 10 min and water for 10 min with continuous stirring. The reaction volume was adjusted for 10 mL with water, and the supernatants recovered after centrifugation at 2500 g for 10 min. The determination was performed using a colorimetric assay using the m-hydroxybiphenyl method, with galacturonic acid as the standard (Filisetti-Cozzi and Carpita, 1991). Four hundred µl from each extraction was incubated with 40μL sulfamic acid 4 M (pH 1.6) and 2.4 mL of sodium tetraborate in sulfuric acid 75 mM. The reactions were incubated at 100 °C in water baths for 20 min, followed by 10 min in ice baths. Eighty µl of m-hydroxydiphenyl of 0.15% (p/v) 3-phenylphenol in sodium hydroxide 5% was added and was read at 525 nm.

### 2.8 Lignin determination

Lignin determination was performed using the protocol by Fukushima and Kerley (2011). Ten milligrams of intact cell walls from leaves were incubated in 250 µL of 25% (v/v) acetyl bromide in glacial acetic acid at 50 °C for 3 h. The material was then centrifuged for 15 min at 10,000 g. One hundred microliters of the extracted material were mixed by inversion with 400 µL of 2M NaOH, 75 µL of 0.5M hydroxylamine hydrochloride, and 1,425 µL of glacial acetic acid. A spectrophotometer measured the colorimetric reaction at 280 nm. The results were normalized by the initial cell wall biomass (10 mg). Lignin content was determined using the equation:


Lignin=(absorbance/(17.75×0.1))×3

### 2.9 Statistical analysis

Growth parameters, cell wall yield, sugar composition, lignin, and uronic content in different CO_2_ and drought treatments were analyzed using ANOVA one-way, followed by Tukey’s test for *post-hoc* comparisons, with a sample size of n = 4. Both tests were performed in R software version 4.2.2 (R Core Team, 2021).

## 3 Results

### 3.1 Identification and expression analysis of transcripts related to cell wall polysaccharide synthesis

The sugarcane variety SP80-3280 features a highly allopolyploid genome (Souza et al., 2019), which presents substantial challenges for genomic and transcriptomic analyses due to the presence of multiple sequence copies arising from extensive hybridization events (Thirugnanasambandam et al., 2018). To assess transcriptional modifications induced by elevated CO_2_ and watering in sugarcane leaves, a total RNA-Seq was obtained, using the Solid Total RNA-Seq kit and sequenced on the Solid 5500xl platform (1x75bp). For sequencing data quality, reads with high-quality scores (Q<20) were considered. Most samples yielded more than 10 million raw reads, except for eCO_2_+Dro (~8 million) (**Supplementary Figure 1**). Great alignment metrics were achieved for sample reads mapped against the reference genome (Souza et al., 2019), with low stringency estimates generally exceeding 80%. This strong concordance between alignment algorithms suggests a limited depth of transcriptome coverage. Additionally, approximately 15% of the reads were estimated to originate from contaminants, including prokaryotes and other eukaryotes present in the samples (**Supplementary Figure 1**).

A total of 36,608 transcripts were identified based on the reference genome. To overcome these complexities, functional annotation was employed to select sequences, which were subsequently characterized to identify domains associated with NDP-sugar interconversion and GTs involved in the synthesis of cell wall polysaccharides. Through this approach, 1,057 sequences were identified across 32 transcript families within the SP80-3280 genome (Souza et al., 2019). Among these, 469 sequences were linked to the NDP-sugar interconversion pathway, while 588 were associated with polysaccharide assembly by GTs (**Table 1**). Comprehensive domain characterizations of these sequences are detailed in **Supplementary Data Sheet 2**.

**Table 1:** Number of sequences in each gene family related to cell wall polysaccharide synthesis identified and expressed in sugarcane (SP80-3280) leaves under different CO_2_ and watering conditions.

Gene Family	Annotated in SP80-3280 genome	Expressed across treatments
NDP-sugar pathway		
*Sucrose Synthase*	42	22
*Invertase*	97	21
*Hexokinase*	33	17
*Phosphoglucose isomerase*	19	19
*Phosphoglucomutase*	18	16
*UDP-glucose pyrophosphorylase*	36	8
*Fructokinase*	17	3
*Mannose phosphate isomerase*	14	0
*Phosphomannomutase*	6	0
*Mannose-1P-guanyltransferase*	17	6
*GDP-mannose-4,6-dehydratase*	4	0
*GDP-fucose synthase*	9	0
*Rhamnose biosynthesis enzyme*	23	8
*UDP-glucose-6-dehydrogenase*	21	11
*UDP-galactose-4-epimerase*	21	14
*UDP-apiose/UDP-xylose synthase*	4	0
*UDP-glucuronate-decarboxylase*	30	8
*Glucuronate-4-epimerase*	12	0
*UDP-arabinose-4-epimerase*	24	0
*UDP-arabinopyranose-mutase*	22	4
**Total**	469	157
Glycosyltransferase		
*Cellulose synthase*	86	44
*Cellulose synthase like F/H*	106	9
*1,4-β-D-xylan synthase Complex*	78	4
*Xylan arabinosyltransferase*	112	9
*Glucuronoxylan glucuronyltransferase*	29	9
*Cellulose synthase-like C*	14	1
*Xyloglucan 6-xylosyltransferase*	26	2
*α-galacturonosyltransferase*	99	13
*Rhamnogalacturonan I rhamnosyltransferase 1*	17	1
*Arabinnan Deficinent Protein*	4	0
*Galactan synthase Synthase*	17	0
**Total**	588	92

The expression analysis was performed on plants grown under varying CO_2_ concentrations and watering conditions.

In the UDP-sugar metabolism pathways (map00520) for generating other NDP-sugars, *rhamnose biosynthesis enzyme* (*RHM*), *UDP-galactose-4-epimerase* (*GALE*), and *UDP-glucose dehydrogenase* (*UGD*) exhibited eight, 14, and two expressed sequences, respectively (**Table 1**, **Supplementary Data Sheet 3**). UDP-glucuronate, produced by UGD, is subsequently converted into UDP-xylose by *UDP-glucuronate decarboxylase* (*UXS*), which showed eight expressed sequences. Additionally, *UDP-arabinopyranose mutase* (*UAM*) exhibited four expressed sequences (**Table 1**, **Supplementary Data Sheet 3**). However, the *UDP-arabinose-4-epimerase* (*UAE*) family, involved in the interconversion of UDP-xylose and UDP-arabinopyranose, did not exhibit any expressed sequences. Similarly, other enzymes such as *Glucuronate-4-Epimerase* (*GAE*), responsible for producing UDP-galacturonic acid, and *UDP-Glucuronate-decarboxylase* (*AXS*), which synthesizes UDP-apiose, also lacked expression in all treatments (**Figure 1**, **Table 1**, **Supplementary Data Sheet 3**).

GDP-mannose and GDP-fucose synthesis operate independently of Glc and relies on an alternative pathway. In this pathway, *Fructokinase* (*FK*) exhibited three expressed sequences, while *Mannose-1P-guanyltransferase* (*MGP*) displayed six expressed sequences (**Table 1**, **Supplementary Data Sheet 3**). However, other enzymes involved in this pathway, including *Mannose Phosphate Isomerase* (*MPI*), *Phosphomannomutase* (*PMM*), *GDP-Mannose-4,6-dehydratase* (*GMD*), and *GDP-Fucose Synthase* (*GER/MUR*), did not show any expression across treatments (**Table 1**, **Supplementary Data Sheet 3**).

In GTs, *Cellulose Synthase* (*CesA*) displayed 44 expressed sequences (**Table 1**, **Supplementary Data Sheet 3**). GTs involved in hemicellulose synthesis in the Golgi complex showed varying expression levels: In GAX assembly, *1,4-β-D-xylan synthase Complex* (*XSC*) had four, *Xylan arabinosyltransferase* (*XAT*) had nine, and *Glucuronoxylan glucuronyltransferase* (*GUX*) also exhibited nine expressed sequences. For MLG synthesis, *Cellulose Synthase-like F* and *H* (*CslF/H*) exhibited nine expressed sequences. XG synthesis families demonstrated lower expression, with *Cellulose synthase-like C* (*CslC*) showing only one expressed sequence and *Xyloglucan 6-xylosyltransferase* (*XXT*) showing two. In pectin synthesis, *α-Galacturonosyltransferase* (*GAUT*), responsible for HG production, displayed 13 expressed sequences. However, transcripts for *Rhamnogalacturonan I rhamnosyltransferase 1* (*RRT*), involved in RG-I synthesis, had only one expressed sequence. Other GTs linked to RG-I synthesis, such as *Arabinan Deficient Protein* (*ARAD1/2*) and *Galactan Synthase* (*GALS1*), also were not expressed.

**Figure 3:**
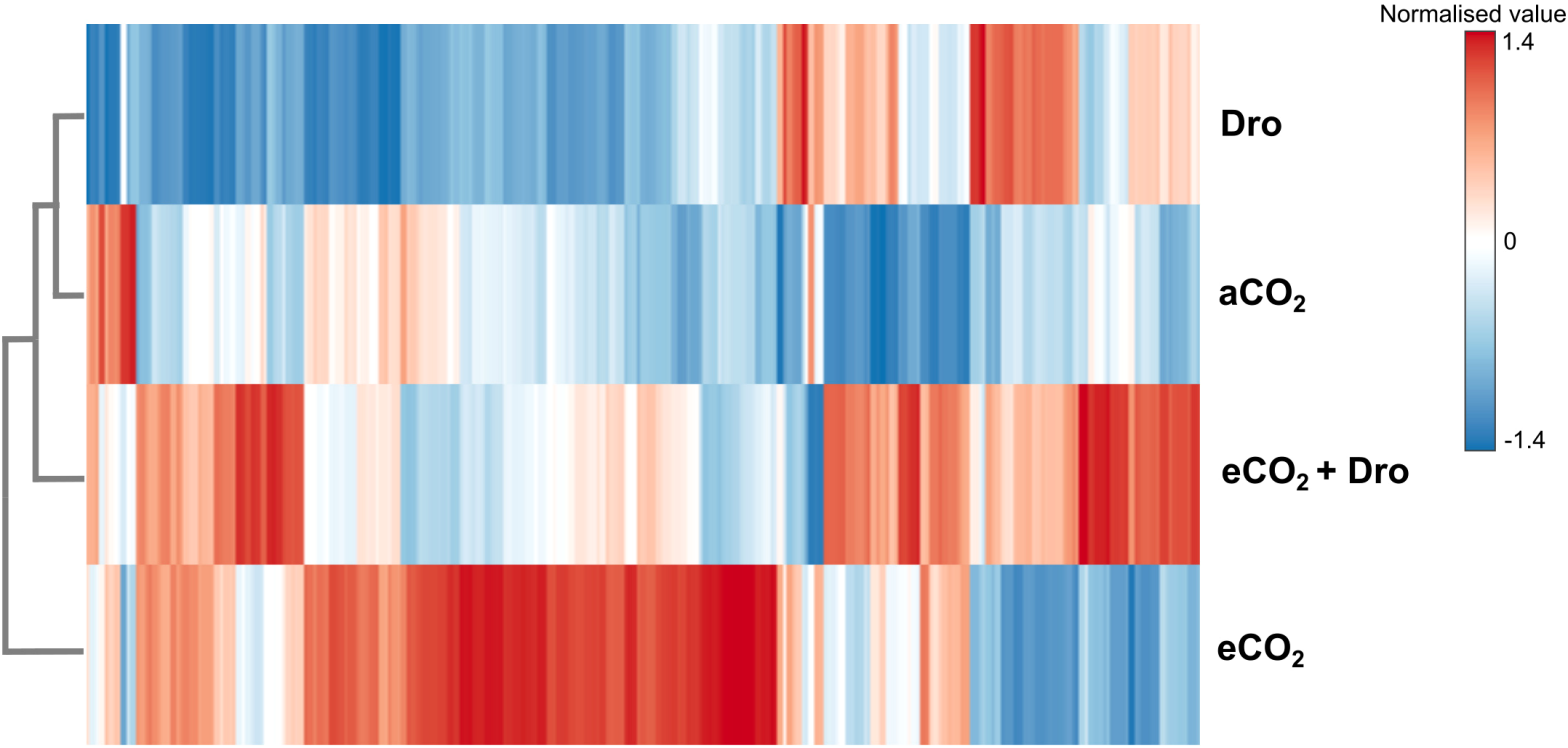
Expression patterns of 32 gene families related to the synthesis of cell wall polysaccharides. Experimental treatments: aCO_2_ (ambient CO_2_, 390 ppm), eCO_2_ (elevated CO_2_, 780 ppm), Dro (drought), and eCO_2_+Dro (elevated CO_2_, 780 ppm, combined with drought).

A great number of sequences were identified in the SP80-3280 genome. However, only 23.5% of these were expressed across the experimental treatments. To understand overall expression among treatments, cluster analysis using all sequences revealed that the expression pattern under aCO_2_ conditions was more like that observed under Dro, whereas eCO_2_+Dro and eCO_2_ exhibited the most distinct expression patterns (**Figure 3**). These results underscore the pronounced impact of eCO_2_ on the expression of transcripts involved in cell wall polysaccharide synthesis within this sequence pool. Furthermore, an antagonistic relationship was observed between eCO_2_ and Dro in expression patterns (**Figure 3**), while eCO_2_+Dro displayed a mixed pattern, combining elements of both eCO_2_ and Dro (**Figure 3**).

Despite the observed relationships, the precise changes induced by the proposed treatments remain unclear. To gain a deeper understanding of these effects, it is essential to pinpoint the specific sequences within each transcript family that are directly impacted. To this end, a multiple comparison analysis between treatments was performed to assess their influence on transcript expression. This analysis identified 69 differentially expressed (DE) sequences across 16 gene families. Notably, most DE sequences exhibited significant expression changes only between contrasting treatments, particularly between eCO_2_ and Dro. In contrast, aCO_2_ and eCO_2_+Dro showed intermediate expression levels. This suggests that the impact of treatments on transcript expression is most pronounced in conditions of environmental contrasts. The focus of the analysis was on sequences with expression levels significantly deviating from the control condition (aCO_2_) to elucidate the specific impacts of treatments on sugarcane cell wall biosynthesis. Comprehensive details of expression differences across treatments are provided in **Supplementary Data Sheet 4**.

**Figure 4:**
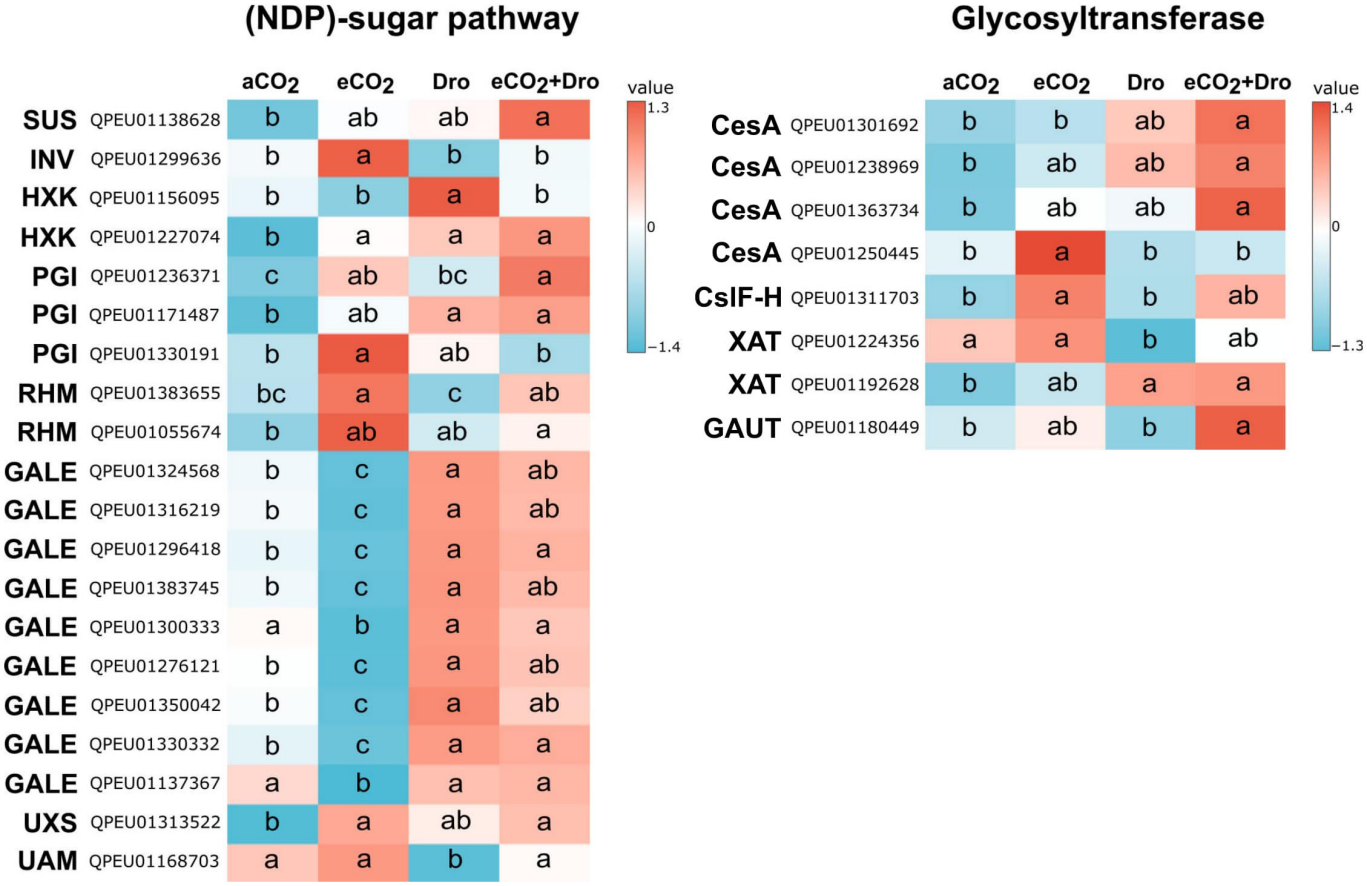
Differential gene expression of cell wall polysaccharide synthesis families in sugarcane SP80-3280 leaves under varying CO_2_ and watering conditions. Differences were identified when any treatment significantly differed from the control condition, with further comparisons among all treatments detailed in **Supplemental Data Sheet 4**. Significant differences (p< 0.05) were determined using a Generalized Linear Model followed by Tukey's test, with different letters indicating significance. Expression values are presented as log_10_ counts per million (CPM), normalized for each sequence. Treatments include aCO_2_ (ambient CO_2_, 390 ppm), eCO_2_ (elevated CO_2_, 780 ppm), Dro (drought), and eCO_2_+Dro (elevated CO_2_, 780 ppm, combined with drought). SUS, sucrose synthase; PGI, phosphoglucose isomerase; INV, invertase; HXK, hexokinase; RHM, rhamnose biosynthesis enzyme; UXS, UDP-glucuronate decarboxylase; GALE, UDP-galactose-4-epimerase; UAM, UDP-arabinopyranose mutase; CesA, cellulose synthase; CslF-H, cellulose synthase-like F-H; XAT, xylan arabinosyltransferase; GAUT, α-galacturonosyltransferase.

To assess expression differences in sequences related to cell wall polysaccharide synthesis, a GLM was applied, and the targets that showed any difference compared to the control (aCO_2_) are highlighted in **Figure 4**. The *SUS*, *INV*, *HXK*, and *PGI* transcript families exhibited DE compared to aCO_2_, with distinct responses under the experimental conditions. The *SUS* family showed 11 DE (**Supplementary Table 4**); however, only the sequence *QPEU01138628* contrasted, exhibiting a 1.7-fold increase under eCO_2_+Dro compared to aCO_2_ (**Figure 4**). Similarly, in the *INV* family, despite eight DE sequences, only *QPEU01299636* differed from aCO_2_, showing a 2.8-fold increase under eCO_2_. Nevertheless, this difference disappeared in the combined treatment (eCO_2_+Dro), indicating a buffering effect (**Figure 4**). The *HXK* family exhibited five DE sequences. The sequence *QPEU01156095* showed a 3.6-fold increase in response to Dro, while *QPEU01227074* was responsive across all conditions, with at least a 2-fold increase under both eCO_2_ and Dro (**Figure 4**). In the *PGI* family, three out of four DE sequences differed from aCO_2_. Notably, *QPEU01236371* and *QPEU01330191* displayed increases of 1.7-fold and 2.2-fold under eCO_2_, respectively, while *QPEU01236371* showed a more pronounced 2.1-fold increase under the combined treatment. Sequence *QPEU01171487* was responsive to Dro, with at least a 1.7-fold increase under both Dro and eCO_2_+Dro (**Figure 4**).

In the *RHM* family, sequences *QPEU01055674* and *QPEU01383655* were responsive to eCO_2_, with 1.7-fold and 1.8-fold increases, respectively. These differences disappeared in the combined treatment (eCO_2_+Dro). The *GALE* family demonstrated nine sequences DE compared to aCO_2_, with two distinct expression patterns. The first group (*QPEU01324568*, *QPEU01316219*, *QPEU01296418*, *QPEU01383745*, *QPEU01276121*, *QPEU01350042*, and *QPEU01330332*) exhibited reduced expression (~2.5-fold) under eCO_2_ and increased expression (~2.4-fold) under Dro, with some sequences showing buffered effects in the combined treatment. In contrast, the second group (*QPEU01137367*, and *QPEU01300333*) showed reduced expression only under eCO_2_, with no response to Dro (**Figure 4**). In the *UXS* family, sequence *QPEU01313522* exhibited increases of 2.6-fold and 2.9-fold under eCO_2_ and eCO_2_+Dro, respectively. In the *UAM* family, sequence *QPEU01168703* was responsive only to Dro, with a 3.6-fold reduction in expression (**Figure 4**).

Among GTs, *CesA* showed 11 sequences DE, of which only four results with DE contrasting with aCO_2_. Sequence *QPEU01250445* showed a 5-fold increase under eCO_2_, while *QPEU01301692*, *QPEU01238969*, and *QPEU01363734* displayed slight increases under Dro, which became statistically significant in the combined treatment (eCO_2_+Dro) with a ~1.7-fold increase (**Figure 4**). In the synthesis of MLG, sequence *QPEU01311703* from the *CslF-H* family showed a 3.3-fold increase under eCO_2_, but this difference disappeared in the combined treatment. In *GAX* assembly, the *XAT* family displayed contrasting responses, with *QPEU01224356* showing a 1.8-fold reduction under Dro, and *QPEU01192628* exhibiting increases of 2.2-fold under eCO_2_ and 2.3-fold under eCO_2_+Dro (**Figure 4**). Finally, in pectin assembly, the *GAUT* family had only one DE sequence, *QPEU01180449*, which showed a 2.7-fold increase under the combined treatment (eCO_2_+Dro) (**Figure 4**).

These findings underscore the intricate regulatory effects of the treatments on cell wall polysaccharide synthesis pathways, which are pivotal for determining the synthesis and deposition of specific cell wall components. As observed in overall expression, eCO_2_ frequently enhances transcript expression. This can be observed in starch and sucrose metabolism, UDP-rhamnose, and UDP-xylose synthesis, MLG, and cellulose assembly. In constraint, the UDP-galactose and arabinosylation of XG can be affected by drought. Additionally, the combined treatment led to buffering or synergistic effects. Altogether, these results show that different regulation patterns exist depending on the stress treatment, with the combination of stresses being a result of the complex interaction of the combined stresses instead of the predominance of one of them.

### 3.2 Plant biomass allocation

**Figure 5:**
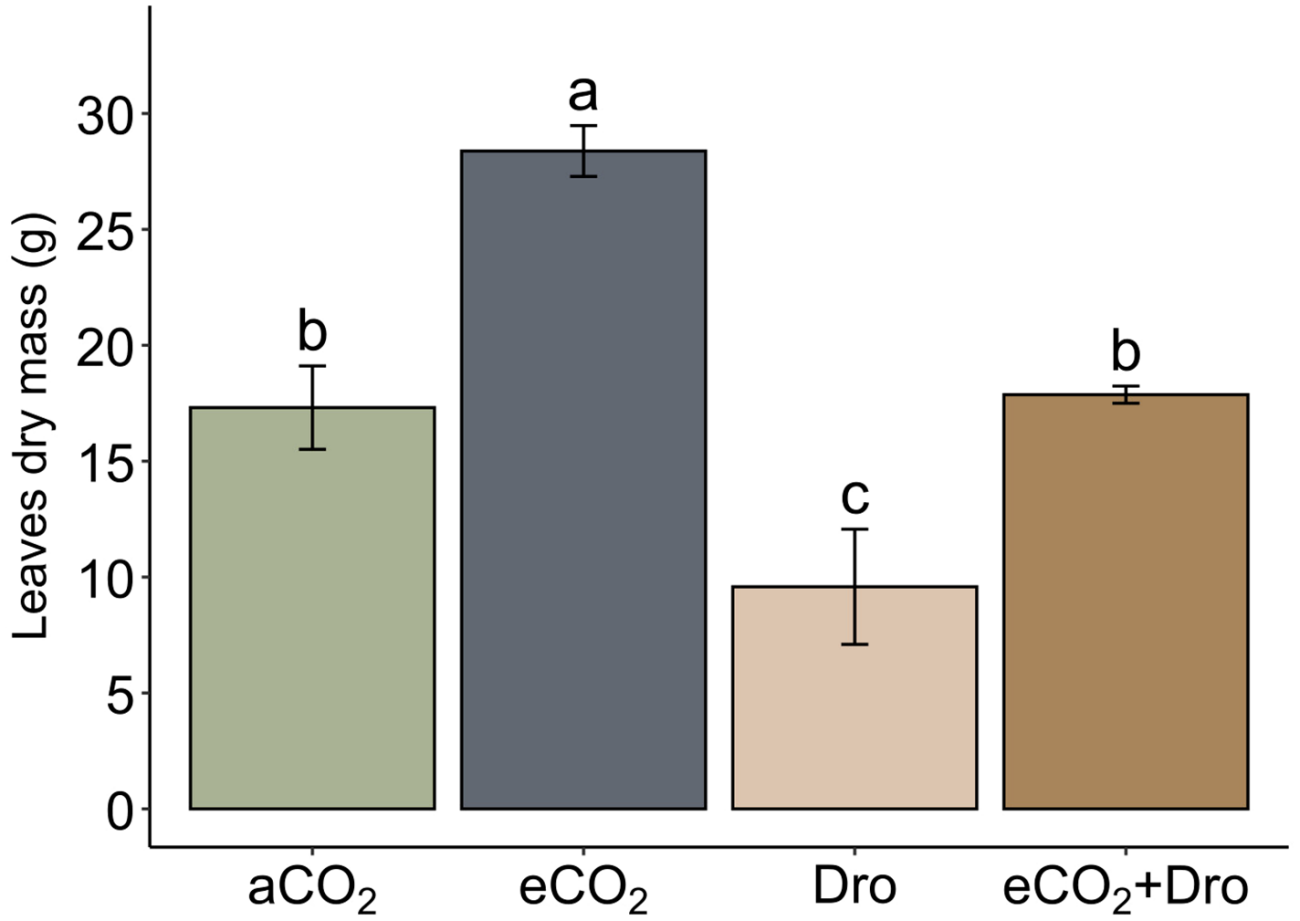
Leaves biomass (dry mass) weight in sugarcane plants grown under different CO_2_ concentrations and watering conditions. Bars represent the mean ± standard error (*n* = 4). Different letters indicate significant differences (p< 0.05), determined by one-way ANOVA and Tukey's test. aCO_2_, ambient CO_2_ (390 ppm); eCO_2_, elevated CO_2_ (780 ppm); Dro, drought; eCO_2_+Dro, elevated CO_2_ (780 ppm) combined with drought.

Sugarcane young plants were grown for 65 days in four treatments [aCO_2_], [eCO_2_], [Dro], and [eCO_2_+Dro] to evaluate the isolated and combined effects of eCO_2_ and drought on productivity. Due to the young age of the plants, the culms represented a tiny part of plant biomass (leaves biomass), while the leaves have at least 92% of biomass in all treatments (**Supplementary Table S1**). Under [eCO_2_], there was a substantial increase of 63.9% (28.4 g) compared to [aCO_2_] (17.3 g) (**Figure 5**). On the other hand, [Dro] resulted in a 44.6% reduction (9.6 g). In the combined treatment [eCO_2_+Dro], leaves biomass (17.9 g) were comparable to that of plants under [aCO_2_] (**Figure 5**), placing it between the effects of [eCO_2_] and [Dro]. These findings highlight the buffering effect exerted by [eCO_2_] on [Dro], which is confirmed in the observation of gene expression described in the item **4.1**.

### 3.3 Structural and non-structural carbohydrates

**Figure 6:**
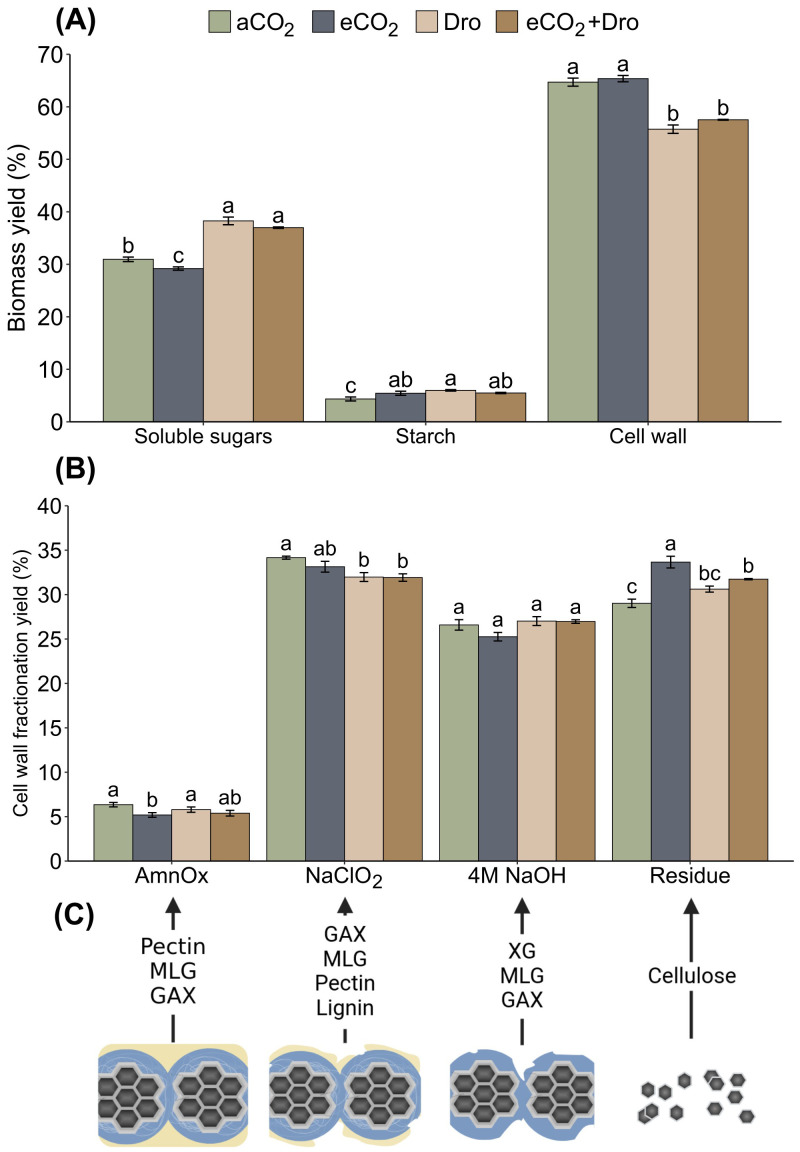
Sugarcane carbohydrate composition of plants grown in different CO_2_ concentrations and watering conditions. **(A)** Alcohol soluble compounds (ASC), starch, and cell wall proportions. **(B)** Yield of cell wall sequential fractionation. **(C)** Cell wall degradation along sequential extraction. The results were expressed in relative form. Bars are represented by mean ± standard error (*n* = 4). Different letters indicate significant differences (p< 0.05), determined by one-way ANOVA and Tukey's test. aCO_2_, ambient CO_2_ (390 ppm); eCO_2_, elevated CO_2_ (780 ppm); Dro, drought; eCO_2_+Dro, elevated CO_2_ (780 ppm) combined with drought; AmnOx, ammonium oxalate fraction; NaClO_2_, sodium chlorite fraction; 4M NaOH, sodium hydroxide fraction; MLG, mixed-linked-glucan; GAX, glucuronoarabinoxylan; and XG, xyloglucan.

The carbohydrate composition in leaves were evaluated as alcohol-soluble compounds (soluble sugars) and starch (as non-structural carbohydrates), and cell wall (structural carbohydrates). The soluble sugars represented 29-38% of biomass, starch around 6%, totalizing about 45% of leaves biomass, and the remainder comprising cell wall with 55-65% (**Figure 6A**). On treatments of [Dro] and [eCO_2_+Dro], soluble sugars increased by approximately 10% whereas the walls were reduced by 14% and 11%, respectively (**Figure 6A**).

**Figure 7:**
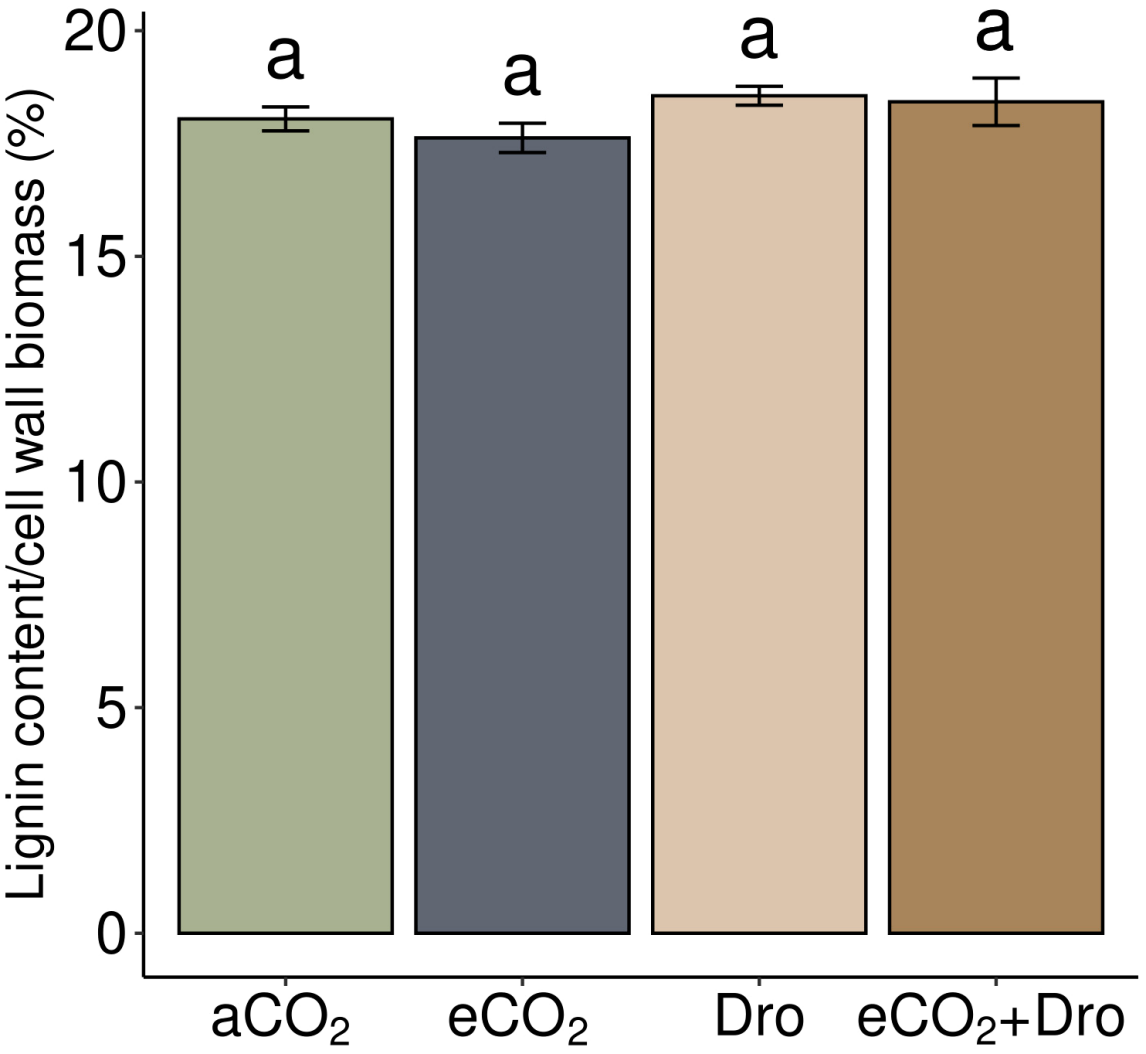
Lignin relative content in sugarcane cell walls under different CO_2_ concentrations and drought. Bars represented by mean ± standard error (n = 4). Different letters indicate significant differences (p< 0.05), determined by one-way ANOVA and Tukey's test. aCO_2_, ambient CO_2_ (390 ppm); eCO_2_, elevated CO_2_ (780 ppm); Dro, drought; eCO_2_+Dro, elevated CO_2_ (780 ppm) combined with drought.

Cell wall components were separated into four distinct solubilized fractions, each characterized by its specific polysaccharide classes. According to the previous characterization of sugarcane leaf cell walls (De Souza et al., 2013), the first fraction (ammonium oxalate fraction, AmmOX) is composed of pectins and some soluble hemicelluloses (**Figure 6C**). This fraction accounted for 9.8% of the total cell wall biomass under [aCO_2_] conditions (**Figure 6B**). Little significant changes were observed in the AmmOX fraction [Dro] and [eCO_2_+Dro] treatments, with a 19.2% reduction in yield being found under [eCO_2_] treatment. The second fraction (sodium chlorite fraction, NaClO_2_) consists of soluble hemicelluloses and some polysaccharides linked to phenylpropanoid chains (**Figure 6C**). The NaClO_2_ fraction under [aCO_2_] accounted for 34.2% of the cell wall biomass, with approximately 52.6% of this fraction (18% of the total cell wall biomass) consisting of lignin, which remained unchanged across treatments (**Figure 7**). Although lignin content did not change, the NaClO_2_ fraction decreased by 6.4% and 6.6% under [Dro] and [eCO_2_+Dro], respectively (**Figure 6B**). The third fraction (4M NaOH fraction, **Figure 6C**), consisting of hemicelluloses strongly adhering to cellulose, accounted for 26.6% of the cell wall biomass, with no significant differences observed across treatments (**Figure 6B**). Finally, the residue fraction (mostly cellulose) comprised 29.4% of the total cell wall biomass. Under [eCO_2_] and [eCO_2_+Dro], the residue fraction increased by 14.3% and 7.8%, respectively. The lignin levels were not affected by any of the treatments (**Figure 7**). Thus, in terms of mass, carbohydrates are affected, mainly soluble sugars and cell walls. In the latter, the most evident changes were related to polymers associated with lignin (NaClO_2_ fraction) and cellulose.

### 3.4 Cell wall neutral monosaccharides and uronic composition

**Figure 8:**
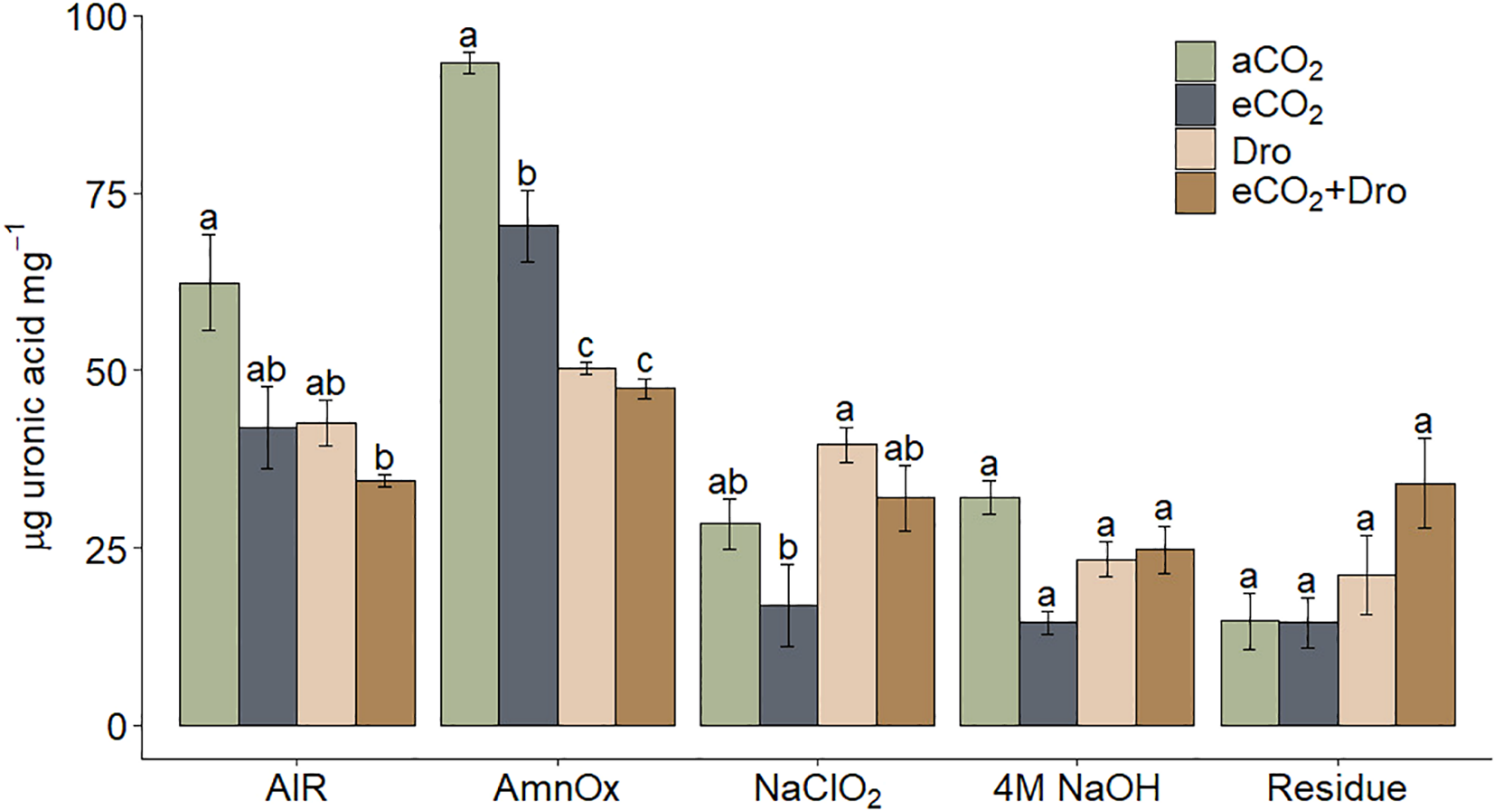
Uronic acid levels in AIR and different fractions of Cell Wall in sugarcane leaves. Bars represented by mean ± standard error (*n* = 4). Different letters indicate significant differences (p< 0.05), determined by one-way ANOVA and Tukey's test. aCO_2_ = ambient CO_2_ (390 ppm), eCO_2_= elevated CO_2_ (780 ppm), Dro = drought, eCO_2_+Dro = elevated CO_2_ (780 ppm) combined with drought. AIR, Alcohol Insoluble Residue (intact cell wall); AmnOx, ammonium oxalate fraction; NaClO_2_, sodium chlorite fraction; 4M NaOH, sodium hydroxide fraction.

The uronic acid analysis represents the cell wall's galacturonic and glucuronic acid content. This was quantified in both AIR and cell wall fractions. In the AIR fraction, uronic acid content was measured at 62 µg.mg^-^¹ of cell wall biomass (**Figure 8**). A slight reduction was observed under [eCO_2_] and [Dro] conditions, though these changes were not statistically significant. A more pronounced decrease of 44.3% was detected under the combined [eCO_2_+Dro] treatment (**Figure 8**). The AmnOX fraction exhibited the highest uronic acid concentration, with 92.4 µg.mg^-^¹ under [aCO_2_] conditions. All three treatments reduced uronic acid content in the AmnOX fraction, with decreases of 28.5% under [eCO_2_], 45.6% under [Dro], and 48.8% under the combined [eCO_2_+Dro] treatment (**Figure 8**). The NaClO_2_ fraction contained 25.7 µg.mg^-^1 of uronic acid, with no statistically significant changes observed under any treatment. Although [eCO_2_] caused a slight reduction and [Dro] a slight increase, neither effect reached statistical significance (**Figure 8**). Similarly, no differences in uronic acid content were detected in the 4M NaOH and Residue fractions under any treatment conditions (**Figure 8**).

The neutral monosaccharide content was similar across treatments, with minor differences indicate changes in polysaccharide composition. In the AmnOX fraction, the most abundant monosaccharides were glucose, xylose, and arabinose (**Table 2**), followed by galactose as the fourth most prevalent monosaccharide. Traces of mannose and rhamnose were also detected, but fucose was absent. A significant difference compared to [aCO_2_] was observed only in glucose content, which decreased by 40.6% under [eCO_2_] conditions.

**Table 2:** Monosaccharide composition in cell wall fractions from sugarcane leaves hydrolyzed with TFA and H_2_SO_4_. Data represented by the mean ± standard error (n =4) in μg.mg^-1^.

Fractions	Monosaccharides	aCO_2_	eCO_2_	Dro	eCO_2_+Dro	*p*-value
AmnOX	Fucose	n.d.	n.d.	n.d.	n.d.	**-**
	Arabinose	6.08 ± 0.44 a	6.28 ± 0.25 a	5.61 ± 0.29 a	5.44 ± 0.35 a	0.308
	Galactose	3.90 ± 0.21 a	4.01 ± 0.27 a	3.39 ± 0.26 a	3.54 ± 0.49 a	0.52
	**Rhamnose**	**1.24 ± 0.10 ab**	**1.49 ± 0.10 a**	**0.91 ± 0.07 b**	**1.01 ± 0.15 b**	**0.013**
	**Glucose**	**8.99 ± 0.48 a**	**5.34 ± 0.31 b**	**11.38 ± 0.93 a**	**10.57 ± 1.01 a**	**0.001**
	Xylose	11.04 ± 1.72 a	11.57 ± 1.03 a	9.71 ± 0.76 a	8.76 ± 0.95 a	0.359
	Mannose	2.01 ± 0.29 a	2.11 ± 0.22 a	2.34 ± 0.17 a	2.19 ± 0.40 a	0.862
NaClO_2_	**Fucose**	**0.65 ± 0.03 b**	**0.77 ± 0.13 b**	**0.86 ± 0.13 ab**	**1.24 ± 0.14 a**	**0.008**
	**Arabinose**	**50.97 ± 3.05 b**	**53.04 ± 6.98 ab**	**53.69 ± 6.98 ab**	**64.99 ± 3.62 a**	**0.02**
	**Galactose**	**9.89 ± 0.39 b**	**8.72 ± 0.65 b**	**10.94 ± 0.65 ab**	**13.35 ± 0.84 a**	**0.009**
	**Rhamnose**	**0.66 ± 0.0 3 b**	**0.71 ± 0.06 b**	**0.71 ± 0.06 b**	**0.91 ± 0.04 a**	**0.013**
	**Glucose**	**37.24 ± 3.1 bc**	**26.50 ± 6.19 c**	**54.98 ± 5.32 ab**	**62.06 ± 4.66 a**	**0.001**
	Xylose	**174.13 ± 10.43 a**	**211.83 ± 2.69 a**	**178.13 ± 10.74 a**	**210.62 ± 10.51 a**	**0.02**
	Mannose	0.42 ± 0.01 a	0.44 ± 0.05 a	0.37 ± 0.03 a	0.36 ± 0.03 a	0.381
4M NaOH	Fucose	0.60 ± 0.4 5 a	0.76 ± 0.37 a	1.27 ± 0.44 a	1.05 ± 0.37 a	0.644
	**Arabinose**	**76.39 ± 5.97 a**	**68.21 ± 4.85 ab**	**59.25 ± 1.44 b**	**62.75 ± 1.49 ab**	**0.049**
	**Galactose**	**10.29 ± 1.19 a**	**7.19 ± 0.46 b**	**8.11 ± 0.14 ab**	**8.47 ± 0.21 ab**	**0.034**
	Rhamnose	1.96 ± 0.36 a	1.30 ± 0.23 a	1.67 ± 0.13 a	1.56 ± 0.16 a	0.312
	Glucose	69.40 ± 6.82 a	54.50 ± 6.04 a	65.97 ± 3.42 a	64.06 ± 1.41 a	0.223
	**Xylose**	**332.25 ± 18.78 a**	**345.33 ± 21.09 a**	**267.04 ± 5.88 a**	**274.39 ± 9.64 a**	**0.032**
	Mannose	5.99 ± 0.88 a	5.83 ± 0.82 a	3.74 ± 0.25 a	4.46 ± 0.45 a	0.088
Residue (TFA)	Fucose	n.d.	n.d.	n.d.	n.d.	-
	Arabinose	2.02 ± 0.05 a	1.77 ± 0.15 a	1.98 ± 0.09 a	1.82 ± 0.07 a	0.239
	**Galactose**	**0.78 ± 0.03 a**	**0.54 ± 0.03 b**	**0.84 ± 0.04 a**	**0.75 ± 0.04 a**	**0.001**
	Rhamnose	n.d.	n.d.	n.d.	n.d.	-
	**Glucose**	**26.78 ± 1.70 b**	**34.18 ± 1.19 a**	**29.11 ± 0.95 b**	**27.99 ± 0.13 b**	**0.003**
	Xylose	3.70 ± 0.10 a	3.52 ± 0.25 a	3.54 ± 0.08 a	3.30 ± 0.13 a	0.396
	Mannose	n.d.	n.d.	n.d.	n.d.	-
Residue (H_2_SO_4_)	Fucose	n.d.	n.d.	n.d.	n.d.	-
	Arabinose	1.53 ± 0.08 a	1.21 ± 0.06 a	1.54 ± 0.07 a	1.15 ± 0.05 a	0.287
	Galactose	0.57 ± 0.01 a	0.35 ± 0.03 a	0.64 ± 0.04 a	0.45 ± 0.02 a	0.259
	Rhamnose	n.d.	n.d.	n.d.	n.d.	-
	Glucose	303.95 ± 16.26 a	294.02 ± 13.25 a	304.61 ± 5.34 a	264.75 ± 7.43 a	0.344
	Xylose	6.82 ± 0.57 a	5.61 ± 0.17 a	7.48 ± 0.15 a	5.92 ± 0.11 a	0.807
	Mannose	1.44 ± 0.12 a	1.21 ± 0.01 a	1.77 ± 0.11 a	1.17 ± 0.09 a	0.36

Differences were determined by one-way ANOVA (p< 0.05) (bold) and letters indicate group separation by Tukey's test. AmnOx, ammonium oxalate fraction; NaClO_2_, sodium chlorite fraction; 4M NaOH, 4M sodium hydroxide fraction; aCO_2_, ambient CO_2_ (390 ppm); eCO_2_, elevated CO_2_ (780 ppm); Dro, drought; eCO_2_+Dro, elevated CO_2_ (780 ppm) combined with drought; n.d., not detected.

In the NaClO_2_ fraction, xylose constituted nearly 50% of the total monosaccharides, with a concentration of 174 µg.mg^-^¹ under [aCO_2_]. Despite the absence of statistical significance due to data variability, a slight increase in xylose content was observed under [eCO_2_], regardless of drought (**Table 2**). Glucose (37.2 µg.mg^-^¹) and arabinose (51 µg.mg^-^¹) were also present in considerable amounts, along with galactose (9.9 µg.mg^-^¹), and small quantities of fucose (0.6 µg.mg^-^¹) and rhamnose (0.7 µg.mg^-^¹). Notably, under the combined [eCO_2_+Dro] treatment, increases were observed in glucose (40%), rhamnose (27.5%), galactose (25.9%), arabinose (21.6%), and fucose (45.6%). Traces of mannose were also detected (**Table 2**).

In the 4M NaOH fraction, xylose (332.2 µg.mg^-^¹) remained the most abundant monosaccharide, accounting for more than double the combined amount of other monosaccharides (**Table 2**). Although ANOVA showed reductions in xylose content under [Dro] and [eCO_2_+Dro], the Tukey test could not distinguish significant differences, suggesting these reductions should be interpreted cautiously. Arabinose (76.4 µg.mg^-^¹) and glucose (69.4 µg.mg^-^¹) were also abundant. Glucose content remained unchanged across treatments, and arabinose content decreased by 22.4% under [eCO_2_] (**Table 2**). Galactose content (10.3 µg.mg^-^¹) was reduced by 30.1% under [Dro] conditions (**Table 2**). Traces of mannose, fucose, and rhamnose were also detected but showed no variation across treatments (**Table 2**).

The residue fraction was hydrolyzed using TFA and H_2_SO_4_. TFA hydrolysis released a considerable amount of glucose (26.8 µg.mg^-^¹), which increased by 21.6% under [eCO_2_] (**Table 2**). Smaller amounts of xylose (3.7 µg.mg^-^¹), arabinose (2 µg.mg^-^¹), and galactose (0.8 µg.mg^-^¹) were also detected. A significant reduction in galactose content (30.8%) was observed under [eCO_2_] (**Table 2**). Fucose, rhamnose, and mannose were not detected. Hydrolysis with H_2_SO_4_ yielded a large amount of glucose (303.9 µg.mg^-^¹), with significant changes across treatments (**Table 2**). Small concentrations of xylose (6.8 µg.mg^-^¹), arabinose (1.5 µg.mg^-^¹), mannose (1.4 µg.mg^-^¹), and galactose (0.6 µg.mg^-^¹) were also detected, but no differences were observed among treatments (**Table 2**). Fucose and rhamnose were not detected in this fraction.

Despite not being directly influenced by eCO_2_, the soluble sugars ratio to cell wall biomass increased under drought conditions. Furthermore, drought promotes a significant reduction in uronic content and arabinose. The effects of eCO_2_ were associated more with a slight reduction in the AmnOx fraction, driven by decreased uronic acid, glucose, and galactose content, along with an increase in the residue fraction, likely due to elevated glucose content not previously extracted. Consistent with gene expression patterns, eCO_2_ was observed to either buffer or amplify the effects of drought in the combined treatment, reflecting the nuanced interplay between these environmental factors.

## 4 Discussion

Brazil's economy relies heavily on agriculture, with sugarcane being one of its most important crops. Sugarcane productivity is directly linked to biomass accumulation, which is significantly influenced by abiotic stress (Chandran et al., 2023). In this study, both drought and eCO_2_ affected leaf biomass, while their combined effects acted as a buffer. Analysis of cell wall biomass, composition, and differential transcript expression revealed that cell wall architecture was only slightly impacted by eCO_2_, drought, and their interaction. Furthermore, while previous studies have examined the impact of eCO_2_ or drought on cell walls in model plants, our research provides the first evidence of their individual and combined effects on sugarcane cell walls, highlighting their role in crop resilience under field conditions and their implications for bioenergy production.

### 4.1 Effects of elevated CO_2_ and drought on leaves biomass accumulation and cell wall proportion

eCO_2_ is well known for enhancing water-use efficiency and increasing carbon fixation through photosynthesis, leading to higher biomass productivity in both in C3 plants, and in C4 plants under drought stress (Ottman et al., 2001; Leakey et al., 2006; Fortirer et al., 2023). In this study, the double of concentration eCO_2_ (780 ppm) increased leaf biomass by 64% (**Figure 1**), reinforcing previous observations and corroborating findings in sugarcane SP80-3280 (De Souza et al., 2008) and *S*. *officinarum* L. cv. CP73-1547 (Vu et al., 2006). In contrast, drought reduced leaf biomass by 45% (**Figure 5**), aligning with observations in other sugarcane varieties under drought stress (Júnior et al., 2019; Chapae et al., 2020). However, when eCO_2_ and drought were applied simultaneously, eCO_2_ mitigated the negative effects of drought, leading to biomass accumulation similar to control conditions (**Figure 5**). This buffering effect, also observed in maize and sorghum (Ripley et al., 2022; Rodrigues et al., 2023), suggests a common response among C4 plants, likely due to increased water-use efficiency and enhanced photosynthetic rates (Wang et al., 2022).

The changes in carbon fixation and allocation partitioning under eCO_2_ and drought (De Souza et al., 2008; Vu and Allen, 2009) suggest potential impacts on cell wall structure. On one hand, the cell wall serves as the primary carbon sink in plants; on the other, it plays a crucial role in plant adaptation by balancing structural integrity with plasticity (Hamann et al., 2018). However, the increase in leaf biomass (**Figure 5**) was not associated with cell wall biomass (**Figure 6A**), suggesting that carbon fixation is allocated to sustain rapid growth without altering the ratio of cell wall biomass to total biomass. This observation contrasts with the two-fold reduction in cell wall thickness reported in sorghum (Watling et al., 2000). Interestingly, under drought, a 14% reduction in cell wall biomass was observed (**Figure 6A**), similar to findings in *Picea mariana* under drought stress (Balducci et al., 2015). Furthermore, the buffering effect of eCO_2_ on leaf biomass under drought was not evident in cell wall biomass accumulation (**Figure 6A**).

These results indicate that carbon allocation to the cell wall affects cell wall biomass only under drought conditions. However, the composition of cell wall polysaccharides plays a crucial role in regulating the mechanical and biochemical properties necessary for plant development (Simões et al., 2020; Delmer et al., 2024). To investigate how the sugarcane cell wall composition responds to both eCO_2_ and drought, we analyzed acid and neutral carbohydrates and assessed the expression of genes related to polysaccharide synthesis.

### 4.2 Biochemical and molecular evidence shows modifications in pectin and MLG under elevated CO_2_

The resilience of cell wall biomass under eCO_2_ was accompanied by slight variations in monosaccharide composition. A reduction in glucose content across the AmnOX, NaClO_2_, and 4M NaOH fractions, although not statistically significant in the latter two (**Table 2**), indicates a decrease in MLG content. Interestingly, MLG degradation has been associated with enhanced plant growth (Kim et al., 2023), potentially explaining the rapid leaf biomass accumulation observed under eCO_2_ (**Figure 5**). The molecular data contrasts with chemical evidence, as a sequence of *CslF-H* being more expressed under eCO_2_ (**Figure 4**). Despite this association, the exact role of MLG within the cell wall remains unclear. Further research on MLG dynamics during cell wall elongation could provide valuable insights.

The pectic matrix in SP80-3280 sugarcane comprises ~10% of the cell wall biomass, most represented by homogalacturonan (HG) (De Souza et al., 2013). A decline in HG content has been linked to decreased cell wall rigidity (Ralet et al., 2008). In this context, the observed 28.5% decrease in uronic acid content in the AmnOX fraction (**Figure 8**) contrasts with observed in *Pfaffia glomerata* (Louback et al., 2021). This suggests that the cell wall may become more flexible, potentially facilitating the rapid growth of plants under eCO_2_. Interestingly, an inhibition in soluble pectin accumulation in *Lycium barbarum* L. storage under eCO_2_ (Liang et al., 2021) corroborated that eCO_2_ can influence pectin accumulation. Additionally, the presence of rhamnose in the 4M NaOH fraction (**Table 2**) and the slight, non-statistical reduction in uronic acid content (**Figure 8**) further support the notion of changes in pectin composition. The reduction in galactose within the 4M NaOH fraction (**Table 2**), combined with a decrease in the expression of *GalE* (involved in UDP-galactose synthesis), suggests a reduction in the galactan branch of RG-I. This reduction could indicate a loss of water-binding capacity in the cell wall, potentially resulting in decreased porosity (Makshakova et al., 2018; Klaassen and Trindade, 2020).

The higher expression in a *CesA* sequence (**Figure 4**) could indicate an increase in cellulose content, but its expression level was still lower compared to other sequences within the same family (**Supplementary Table 3**). For this reason, under these conditions, these sequences may be irrelevant to cellulose synthesis, resulting in unchanged glucose observed in the H_2_SO_4_ fraction (cellulose) (**Table 2**). A similar lack of difference was previously observed in the same sugarcane variety and developmental stage by De Souza et al. (2008).

### 4.3 Cell wall composition changes in drought combined or not with elevated CO_2_

Cell wall loosening is frequently associated with abiotic stress responses, and the reduction in the cell wall-to-biomass ratio may reinforce this modification under drought stress (**Figure 6**). In this context, the observed differences in pectin composition suggest potential cell wall remodeling. The significant 46% decrease in uronic acid content in the AmnOx fraction highlights the relationship between pectin degradation and drought conditions, consistent with findings in sensitive wheat and maize cultivars (Liu et al., 2021; Calderone et al., 2024). Additionally, a slight increase in rhamnose, and galactose in the NaClO_2_ fraction (**Table 1**) may indicate an increase in RG-I branching, that is boosted by eCO_2_ exposure (**Table 1**). This enhanced branching degree has also been observed in drought-tolerant wheat and was associated with improved cell hydration, mitigating damage under drought conditions (Leucci et al., 2008).

The substantial increase in *GALE* expression (**Figure 4**) under drought and combined reinforces the observed rise in galactose content within the cell wall. However, it is important to note that UDP-galactose is also a precursor for raffinose, a well-documented sugar involved in drought stress responses.

In the hemicellulose domain, the effects of drought suggest modifications in MLG and GAX, while the combination of both stresses resulted in changes in XG. Under drought, a reduction in arabinose and xylose content in the 4M NaOH fraction (**Table 2**), along with a slight decrease in uronic acid content (**Figure 8**), and the downregulation of *XAT* (xylan arabinosyl transferase) and *UAM* (UDP-arabinofuranose mutase) (**Figure 4**), suggests a reduction in GAX (glucuronoarabinoxylan) under drought conditions. These findings support the hypothesis that modifications in arabinoxylan structure can enhance drought resistance (Barbut et al., 2024; Shrestha et al., 2019). Additionally, the increased glucose content in the NaClO_2_ fraction could indicate an expansion of MLG chains, a phenomenon previously associated with drought and heat stress (Rakszegi et al., 2014). However, the limited understanding of MLG function prevents definitive conclusions about its role in stress responses. These compositional adjustments highlight the nuanced role of cell wall modifications in plant adaptation to combined eCO_2_ and drought stress.

### 4.4 Reflections on cell wall responses to drought, eCO_2_, and bioenergy

**Figure 9:**
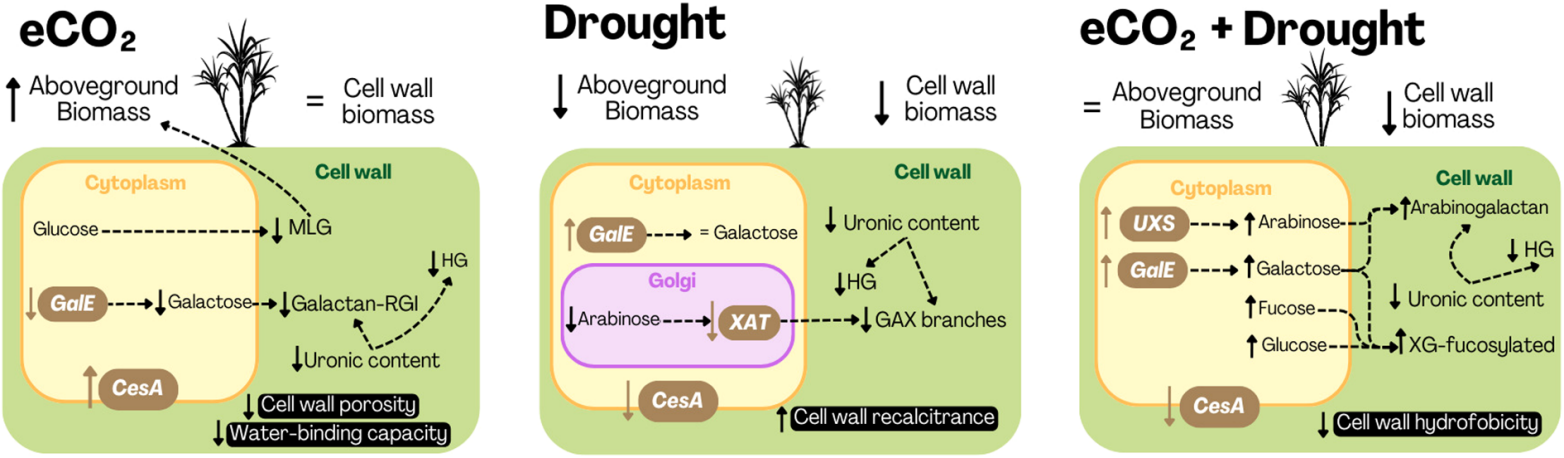
Summary of results and proposed interpretations (black rectangle) of the impact of isolated and combined elevated CO_2_ (eCO_2_) and drought on cell wall synthesis and composition in sugarcane SP80-3280 leaves. UXS, UDP-glucuronate decarboxylase; GalE, UDP-galactose-4-epimerase; UAM, UDP-arabinopyranose mutase; CesA, cellulose synthase; XAT, xylan arabinosyltransferase; GAX, glucuronoarabinoxylan; HG, homogalacturonan; MLG, mixed-glucan; RG, rhamnogalacturonan; XG, xyloglucan.

The results and findings of this study, along with the proposed interpretations, are summarized in **Figure 9**. These findings highlight the relatively minor alterations in monosaccharide composition, while emphasizing the crucial roles of pectin and hemicellulose modifications in plant stress responses. These changes influence the physical and chemical properties of the cell wall, promoting slight adaptations that ensure plant development under unfavorable conditions.

The increased recalcitrance of the cell wall, as indicated by pectin content and the arabinosylation of GAX under drought, can directly influence the conversion of cell wall sugars into biofuels (Xiao and Anderson, 2013). Pectin limits enzymatic access to other cell wall components, thereby hindering hydrolysis during the saccharification process. The reduction in arabinosylation of GAX may, on one hand, strengthen its interactions with cellulose, while on the other, it could reduce lignin binding sites and alter the dynamics of xylan-lignin interactions, which may either promote or hinder cell wall hydrolysis. Conversely, the degradation of MLG under elevated CO_2_ conditions appears to enhance biomass accessibility for industrial processing by facilitating enzymatic access and reducing barriers to conversion. Furthermore, the retention of cellulose content suggests that the yield of fermentable sugars may be sustained, even under challenging environmental conditions. These findings underscore the significance of understanding how cell wall modifications impact biomass conversion efficiency, providing valuable insights for improving bioenergy production strategies.

## 5 Conclusion

This study demonstrates the contrasting effects of eCO_2_ and drought stress on sugarcane growth and cell wall dynamics. While eCO_2_ increased leaf biomass and buffered the negative effects of drought on overall growth, this protection did not extend to cell wall biomass. Under eCO_2_, glucose and uronic acid reductions suggest changes in MLG and pectin composition, potentially enhancing cell wall flexibility and supporting rapid growth. In contrast, drought reduced arabinosylation of GAX and significantly decreased uronic acid, likely reducing cell wall flexibility and contributing to drought sensitivity. Combined eCO_2_ and drought amplified specific alterations in XG and MLG, highlighting their roles in stress adaptation. The cell wall exhibited notable stability despite these changes, reflecting its structural conservation under stress. These insights emphasize the importance of cell wall plasticity in stress resilience and suggest targets for improving sugarcane productivity under climate change.

## Data Availability

The transcriptome information presented in this study can be found in the Sequence Read Archive (SRA • NCBI) at https://www.ncbi.nlm.nih.gov/sra, under the BioProject accession number PRJNA1138658. The dataset presented is available in the Supplementary Material section.
