# Supplementary material for: Cell wall modulation by drought and elevated CO2 in sugarcane leaves
Source: Front Plant Sci. 2025 Apr 30;16:1567201. doi: 10.3389/fpls.2025.1567201 (PMC12075542; doi:10.3389/fpls.2025.1567201)
Supplement: Supplementary file 1 [file SupplementaryFile1.docx]

***Supplementary Material***

**Supplementary File 1.** Reference list using in Supplementary Data Sheet S1

Anders, N., Wilson, L. F. L., Sorieul, M., Nikolovski, N., & Dupree, P. (2023). β-1, 4-Xylan backbone synthesis in higher plants: How complex can it be?. Frontiers in Plant Science, 13, 1076298. doi: 10.3389/fpls.2022.1076298.

Bonin, C. P., & Reiter, W. D. (2000). A bifunctional epimerase‐reductase acts downstream of the MUR1 gene product and completes the de novo synthesis of GDP‐L‐fucose in Arabidopsis. The Plant Journal, 21(5), 445-454. doi: 10.1046/j.1365-313x.2000.00698.x.

Bonin, C. P., Freshour, G., Hahn, M. G., Vanzin, G. F., & Reiter, W. D. (2003). The GMD1 and GMD2 genes of Arabidopsis encode isoforms of GDP-D-mannose 4, 6-dehydratase with cell type-specific expression patterns. Plant physiology, 132(2), 883-892. doi: 10.1104/pp.103.022368.

Cavalier, D. M., & Keegstra, K. (2006). Two xyloglucan xylosyltransferases catalyze the addition of multiple xylosyl residues to cellohexaose. Journal of Biological Chemistry, 281(45), 34197-34207. doi: 10.1074/jbc.M606379200.

Chen, C., Zhao, X., Wang, X., Wang, B., Li, H., Feng, J., & Wu, A. (2021). Mutagenesis of UDP‐xylose epimerase and xylan arabinosyl‐transferase decreases arabinose content and improves saccharification of rice straw. Plant Biotechnology Journal, 19(5), 863. doi: 10.1111/pbi.13552.

Chou, Y. H., Pogorelko, G., & Zabotina, O. A. (2012). Xyloglucan xylosyltransferases XXT1, XXT2, and XXT5 and the glucan synthase CSLC4 form Golgi-localized multiprotein complexes. Plant physiology, 159(4), 1355-1366. doi: 10.1104/pp.112.199356.

Conklin, P. L., Norris, S. R., Wheeler, G. L., Williams, E. H., Smirnoff, N., & Last, R. L. (1999). Genetic evidence for the role of GDP-mannose in plant ascorbic acid (vitamin C) biosynthesis. Proceedings of the National Academy of Sciences, 96(7), 4198-4203. doi: 10.1073/pnas.96.7.4198.

Damari-Weissler, H., Kandel-Kfir, M., Gidoni, D., Mett, A., Belausov, E., & Granot, D. (2006). Evidence for intracellular spatial separation of hexokinases and fructokinases in tomato plants. Planta, 224, 1495-1502. doi: 10.1007/s00425-006-0387-9.

Decker, D., Aubert, J., Wilczynska, M., & Kleczkowski, L. A. (2023). Exploring redox modulation of plant UDP-glucose pyrophosphorylase. International Journal of Molecular Sciences, 24(10), 8914. doi: 10.3390/ijms24108914.

Degenhardt, J., Poppe, A., Montag, J., & Szankowski, I. (2006). The use of the phosphomannose-isomerase/mannose selection system to recover transgenic apple plants. Plant Cell Reports, 25, 1149-1156. doi: 10.1007/s00299-006-0179-5.

Doblin, M. S., Pettolino, F. A., Wilson, S. M., Campbell, R., Burton, R. A., Fincher, G. B., ... & Bacic, A. (2009). A barley cellulose synthase-like CSLH gene mediates (1, 3; 1, 4)-β-D-glucan synthesis in transgenic Arabidopsis. Proceedings of the National Academy of Sciences, 106(14), 5996-6001. doi: 10.1073/pnas.0902019106.

Du, J., Kirui, A., Huang, S., Wang, L., Barnes, W. J., Kiemle, S. N., ... & Xiao, C. (2020). Mutations in the pectin methyltransferase QUASIMODO2 influence cellulose biosynthesis and wall integrity in Arabidopsis. Plant Cell, 32(11), 3576-3597. doi: 10.1105/tpc.20.00252.

Gallinari, R. H., Coletta, R. D., Araújo, P., Menossi, M., & Nery, M. F. (2020). Bringing to light the molecular evolution of GUX genes in plants. Genetics and molecular biology, 43(1), e20180208. doi: 10.1590/1678-4685-GMB-2018-0208.

Gu, X., Glushka, J., Yin, Y., Xu, Y., Denny, T., Smith, J., ... & Bar-Peled, M. (2010). Identification of a bifunctional UDP-4-keto-pentose/UDP-xylose synthase in the plant pathogenic bacterium Ralstonia solanacearum strain GMI1000, a distinct member of the 4, 6-dehydratase and decarboxylase family. Journal of Biological Chemistry, 285(12), 9030-9040. doi: 10.1074/jbc.M109.066803.

Guyett, P., Glushka, J., Gu, X., & Bar-Peled, M. (2009). Real-time NMR monitoring of intermediates and labile products of the bifunctional enzyme UDP-apiose/UDP-xylose synthase. Carbohydrate research, 344(9), 1072-1078. doi: 10.1016/j.carres.2009.03.026.

Hayashi, T., Koyama, T., & Matsuda, K. (1988). Formation of UDP-xylose and xyloglucan in soybean Golgi membranes. Plant physiology, 87(2), 341-345. doi: 10.1104/pp.87.2.341.

Harholt, J., Jensen, J. K., Sørensen, S. O., Orfila, C., Pauly, M., & Scheller, H. V. (2006). ARABINAN DEFICIENT 1 is a putative arabinosyltransferase involved in biosynthesis of pectic arabinan in Arabidopsis. Plant physiology, 140(1), 49-58. doi: 10.1104/pp.105.072744.

Harholt, J., Jensen, J. K., Verhertbruggen, Y., Søgaard, C., Bernard, S., Nafisi, M., ... & Scheller, H. V. (2012). ARAD proteins associated with pectic Arabinan biosynthesis form complexes when transiently overexpressed in planta. Planta, 236, 115-128. doi: 10.1007/s00425-012-1592-3.

Harper, A. D., & Bar-Peled, M. (2002). Biosynthesis of UDP-xylose. Cloning and characterization of a novel Arabidopsis gene family, UXS, encoding soluble and putative membrane-bound UDP-glucuronic acid decarboxylase isoforms. Plant physiology, 130(4), 2188-2198. doi: 10.1104/pp.009654.

Jang, J. C., Leon, P., Zhou, L., & Sheen, J. (1997). Hexokinase as a sugar sensor in higher plants. The Plant Cell, 9(1), 5-19. doi: 10.1105/tpc.9.1.5.

Kärkönen, A., & Fry, S. C. (2006). Novel characteristics of UDP-glucose dehydrogenase activities in maize: non-involvement of alcohol dehydrogenases in cell wall polysaccharide biosynthesis. Planta, 223, 858-870. doi: 10.1007/s00425-005-0207-7.

Kim, S. J., Chandrasekar, B., Rea, A. C., Danhof, L., Zemelis-Durfee, S., Thrower, N., ... & Keegstra, K. (2020). The synthesis of xyloglucan, an abundant plant cell wall polysaccharide, requires CSLC function. Proceedings of the National Academy of Sciences, 117(33), 20316-20324. doi: 10.1073/pnas.2007245117.

Konishi, T., Aohara, T., Igasaki, T., Hayashi, N., Miyazaki, Y., Takahashi, A., ... & Ishii, T. (2011). Down-regulation of UDP-arabinopyranose mutase reduces the proportion of arabinofuranose present in rice cell walls. Phytochemistry, 72(16), 1962-1968. doi: 10.1016/j.phytochem.2011.07.012.

Konishi, T., Takeda, T., Miyazaki, Y., Ohnishi-Kameyama, M., Hayashi, T., O'Neill, M. A., & Ishii, T. (2007). A plant mutase that interconverts UDP-arabinofuranose and UDP-arabinopyranose. Glycobiology, 17(3), 345-354. doi: 10.1093/glycob/cwl081.

Lee, C., Teng, Q., Zhong, R., & Ye, Z. H. (2012). Arabidopsis GUX proteins are glucuronyltransferases responsible for the addition of glucuronic acid side chains onto xylan. Plant and cell physiology, 53(7), 1204-1216. doi: 10.1093/pcp/pcs064.

Li, J., Chen, G., Zhang, J., Shen, H., Kang, J., Feng, P., ... & Hu, Z. (2020). Suppression of a hexokinase gene, SlHXK1, leads to accelerated leaf senescence and stunted plant growth in tomato. Plant Science, 298, 110544. doi:/10.1016/j.plantsci.2020.110544.

Liu, H. C., Chen, H. C., Huang, T. H., Lue, W. L., Chen, J., & Suen, D. F. (2023). Cytosolic phosphoglucose isomerase is essential for microsporogenesis and embryogenesis in Arabidopsis. Plant Physiology, 191(1), 177-198. doi:/10.1093/plphys/kiac494.

Liwanag, A. J. M., Ebert, B., Verhertbruggen, Y., Rennie, E. A., Rautengarten, C., Oikawa, A., ... & Scheller, H. V. (2012). Pectin biosynthesis: GALS1 in Arabidopsis thaliana is a β-1, 4-galactan β-1, 4-galactosyltransferase. The Plant Cell, 24(12), 5024-5036. doi: 10.1105/tpc.112.106625.

Lugassi, N., Stein, O., Egbaria, A., Belausov, E., Zemach, H., Arad, T., ... & Carmi, N. (2022). Sucrose synthase and fructokinase are required for proper meristematic and vascular development. Plants, 11(8), 1035. doi: 10.3390/plants11081035.

Malinova, I., Kunz, H. H., Alseekh, S., Herbst, K., Fernie, A. R., Gierth, M., & Fettke, J. (2014). Reduction of the cytosolic phosphoglucomutase in Arabidopsis reveals impact on plant growth, seed and root development, and carbohydrate partitioning. PloS one, 9(11), e112468. doi: 10.1371/journal.pone.0112468.

Manoochehri, H., Hosseini, N. F., Saidijam, M., Taheri, M., Rezaee, H., & Nouri, F. (2020). A review on invertase: Its potentials and applications. Biocatalysis and Agricultural Biotechnology, 25, 101599. doi: 10.1016/j.bcab.2020.101599.

Mølhøj, M., Verma, R., & Reiter, W. D. (2003). The biosynthesis of the branched‐chain sugar d‐apiose in plants: functional cloning and characterization of a UDP‐d‐apiose/UDP‐d‐xylose synthase from Arabidopsis. The Plant Journal, 35(6), 693-703. doi: 10.1046/j.1365-313x.2003.01841.x.

Mølhøj, M., Verma, R., & Reiter, W. D. (2004). The biosynthesis of D-Galacturonate in plants. functional cloning and characterization of a membrane-anchored UDP-D-Glucuronate 4-epimerase from Arabidopsis. Plant physiology, 135(3), 1221-1230. doi: 10.1104/pp.104.043745.

Oesterhelt, C., Schnarrenberger, C., & Gross, W. (1997). The reaction mechanism of phosphomannomutase in plants. FEBS letters, 401(1), 35-37. doi: 10.1016/S0014-5793(96)01425-1.

Preiser, A. L., Banerjee, A., Weise, S. E., Renna, L., Brandizzi, F., & Sharkey, T. D. (2020). Phosphoglucoisomerase is an important regulatory enzyme in partitioning carbon out of the Calvin-Benson cycle. Frontiers in Plant Science, 11, 580726. doi: 10.3389/fpls.2020.580726.

Qian, W., Yu, C., Qin, H., Liu, X., Zhang, A., Johansen, I. E., & Wang, D. (2007). Molecular and functional analysis of phosphomannomutase (PMM) from higher plants and genetic evidence for the involvement of PMM in ascorbic acid biosynthesis in Arabidopsis and Nicotiana benthamiana. The Plant Journal, 49(3), 399-413. doi: 10.1111/j.1365-313X.2006.02967.x.

Seifert, G. J. (2004). Nucleotide sugar interconversions and cell wall biosynthesis: how to bring the inside to the outside. Current opinion in plant biology, 7(3), 277-284. doi: 10.1016/j.pbi.2004.03.004.

Stein, O., & Granot, D. (2019). An overview of sucrose synthases in plants. Frontiers in plant science, 10, 95. doi: 10.3389/fpls.2019.00095.

Taketa, S., Yuo, T., Tonooka, T., Tsumuraya, Y., Inagaki, Y., Haruyama, N., ... & Jobling, S. A. (2012). Functional characterization of barley betaglucanless mutants demonstrates a unique role for CslF6 in (1, 3; 1, 4)-β-D-glucan biosynthesis. Journal of experimental botany, 63(1), 381-392. doi: 10.1093/jxb/err285.

Uehara, Y., Tamura, S., Maki, Y., Yagyu, K., Mizoguchi, T., Tamiaki, H., ... & Ishimizu, T. (2017). Biochemical characterization of rhamnosyltransferase involved in biosynthesis of pectic rhamnogalacturonan I in plant cell wall. Biochemical and biophysical research communications, 486(1), 130-136. doi: 10.1016/j.bbrc.2017.03.012.

Usadel, B., Schlüter, U., Mølhøj, M., Gipmans, M., Verma, R., Kossmann, J., ... & Pauly, M. (2004). Identification and characterization of a UDP-D-glucuronate 4-epimerase in Arabidopsis. FEBS letters, 569(1-3), 327-331. doi: 10.1016/j.febslet.2004.06.005.

Watt, G., Leoff, C., Harper, A. D., & Bar-Peled, M. (2004). A bifunctional 3, 5-epimerase/4-keto reductase for nucleotide-rhamnose synthesis in Arabidopsis. Plant Physiology, 134(4), 1337-1346. doi: 10.1104/pp.103.037192.

Williamson, R. E., Burn, J. E., & Hocart, C. H. (2001). Cellulose synthesis: mutational analysis and genomic perspectives using Arabidopsis thaliana. Cellular and Molecular Life Sciences CMLS, 58, 1475-1490. doi: 10.1007/PL00000790.

Zabotina, O. A., Van De Ven, W. T., Freshour, G., Drakakaki, G., Cavalier, D., Mouille, G., ... & Raikhel, N. V. (2008). Arabidopsis XXT5 gene encodes a putative α‐1, 6‐xylosyltransferase that is involved in xyloglucan biosynthesis. The Plant Journal, 56(1), 101-115. doi: 10.1111/j.1365-313X.2008.03580.x.

Zhang, Z., Islam, M. S., Xia, J., Feng, X., Noman, M., Wang, J., ... & Wang, J. (2024). The nucleolin MoNsr1 plays pleiotropic roles in the pathogenicity and stress adaptation in the rice blast fungus Magnaporthe oryzae. Frontiers in Plant Science, 15, 1482934. doi: 10.3389/fpls.2024.1482934.

Zhong, R., Cui, D., Phillips, D. R., Sims, N. T., & Ye, Z. H. (2021). Functional analysis of GT61 glycosyltransferases from grass species in xylan substitutions. Planta, 254(6), 131. doi: 10.1007/s00425-021-03794-y.

**
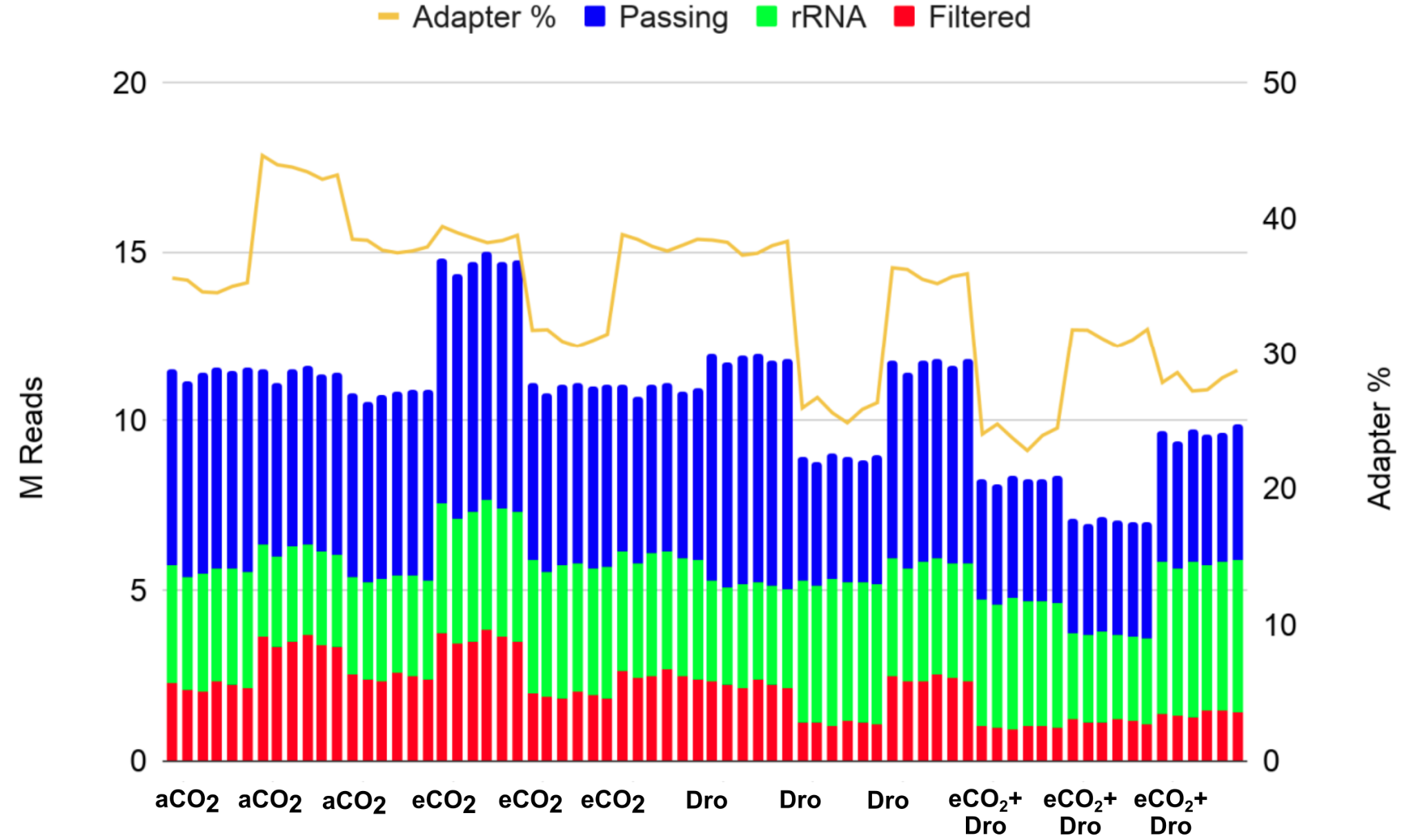
**

**Supplemental Figure S1.** Quantities of reads for each technical replicate (in millions of reads; left axis) after passing through filtering steps: passing (blue), filtered by adapter cleaning and quality control (red), and ribosomal RNA (rRNA; green). The line (yellow; percentage with adapter; right axis) indicates the proportion of reads with detected adapter sequences.

**Supplemental Table S1.** Biomass accumulation in leaves and Culms of sugarcane after 65 days of growth under varying concentrations of CO_2_ and watering conditions. Data are presented as mean biomass (grams) ± standard error (n = 4). Significant differences were determined by ANOVA one-way and Tukey's test (p < 0.05). aCO_2_ = ambient CO_2_ (390 ppm), eCO_2_ = elevated CO_2_ (780 ppm), Dro = drought, eCO_2_+Dro = elevated CO_2_ (780 ppm) combined with drought.

| **Treatment** | **Leaves** | **Culms** |
| --- | --- | --- |
| aCO2 | **b** 17.31g ± 1.80 | **b** 0.47 ± 0.06 |
| eCO2 | **a** 28.38g ± 2.48 | **a** 0.73 ± 0.14 |
| Dro | **c** 9.58g ± 1.10 | **a** 0.71 ± 0.18 |
| eCO2+Dro | **b** 17.87g ± 0.37 | **a** 0.89 ± 0.11 |
